# Endocannabinoid system upregulates the enrichment and differentiation of human iPSC- derived spermatogonial stem cells via CB2R agonism

**DOI:** 10.1186/s40659-025-00596-4

**Published:** 2025-03-12

**Authors:** Merve Gizer, Selin Önen, Özgür Doğuş Erol, Fatima Aerts-Kaya, Tuba Reçber, Emirhan Nemutlu, Petek Korkusuz

**Affiliations:** 1 Department of Stem Cell Sciences, Graduate School of Health Sciences, Hacettepe University, Ankara, 06100 Turkey; 2 METU MEMS Center, Ankara, 06530 Turkey; 3 Center for Stem Cell Research and Development (PEDI-STEM), Hacettepe University, Ankara, 06100 Turkey; 4 Hacettepe University Advanced Technologies Application and Research Center (HÜNİTEK), Ankara, Turkey; 5 Hacettepe University Laboratory Animals Research and Research Center (HÜDHAM), Ankara, Turkey; 6 Department of Analytical Chemistry, Faculty of Pharmacy, Hacettepe University, Sıhhiye, Ankara, 06100 Turkey; 7 Department of Histology and Embryology, Faculty of Medicine, Hacettepe University, Sihhiye, Ankara, 06100 Turkey

**Keywords:** Human induced pluripotent stem cells, Human spermatogonial stem cells, Endocannabinoid systems, CB2R, CB65, Male infertility

## Abstract

**Background:**

Male factor infertility (MFI) is responsible for 50% of infertility cases and in 15% of the cases sperm is absent due to germ cell aplasia. Human induced pluripotent stem cell (hiPSC)-derived spermatogonial stem cells (hSSCs) could serve as an autologous germ cell source for MFI in patients with an insufficient sperm yield for assisted reproductive technology (ART). The endocannabinoid system (ECS) has been implicated to play a role in mouse embryonic stem cells (mESCs) and the human testicular environment. However, the contribution of the ECS in hiPSCs and hiPSC-derived hSSCs is currently unknown. Here, we aimed to assess whether hiPSCs and hiPSC-derived hSSCs are regulated by components of the ECS and whether manipulation of the ECS could increase the yield of hiPSC-derived SSCs and serve as an autologous cell-based source for treatment of MFI.

**Methods:**

We reprogrammed human dermal fibroblasts (hDFs) to hiPSCs, induced differentiation of hSSC from hiPSCs and evaluated the presence of ECS ligands (AEA, 2-AG) by LC/MS, receptors (CB1R, CB2R, TRPV1, GPR55) by qPCR, flow cytometry and immunofluorescent labeling. We then examined the efficacy of endogenous and synthetic selective ligands (ACPA, CB65, CSP, ML184) on proliferation of hiPSCs using real-time cell analysis (RTCA) and assessed the effects of on CB2R agonism on hiPSC pluripotency and differentiation to hSSCs.

**Results:**

hiPSCs from hDFs expressed the pluripotency markers OCT4, SOX2, NANOG, SSEA4 and TRA-1-60; and could be differentiated into ID4+, PLZF + hSSCs. hiPSCs and hiPSC-derived hSSCs secreted AEA and 2-AG at 10^− 10^ − 10^− 9^ M levels. Broad expression of all ECS receptors was observed in both hiPSCs and hiPSC-derived hSSCs, with a higher CB2R expression in hSSCs in comparison to hiPSCs. CB2R agonist CB65 promoted proliferation and differentiation of hiPSCs to hiPSC-hSSCs in comparison to AEA, 2-AG, ACPA, CSP and ML184. The EC_50_ of CB65 was determined to be 2.092 × 10^− 8^ M for support of pluripotency and preservation of stemness on hiPSCs from 78 h. CB65 stimulation at EC_50_ also increased the yield of ID4 + hSSCs, PLZF + SSPCs and SCP3 + spermatocytes from day 10 to 12.

**Conclusions:**

We demonstrated here for the first time that stimulation of CB2R results in an increased yield of hiPSCs and hiPSC-derived hSSCs. CB65 is a potent CB2R agonist that can be used to increase the yield of hiPSC-derived hSSCs offering an alternative source of autologous male germ cells for patients with MFI. Increasing the male germ/stem cell pool by CB65 supplementation could be part of the ART-associated protocols in MFI patients with complete germ cell aplasia.

**Supplementary Information:**

The online version contains supplementary material available at 10.1186/s40659-025-00596-4.

## Background

One in six people at reproductive age experiences infertility according to WHO in 2023 [1]. Male factor infertility (MFI) accounts for approximately 50% and 10–15% of the patients have complete absence of sperm in their ejaculate due to congenital or acquired germ cell aplasia [2, 3]. The assisted reproductive technologies (ART) preserve a small number of spermatogonial stem cells (SSCs) from frozen TESE materials but allow only 54–62% sperm retrieval in azoospermia patients [4–6]. Experimental in vitro spermatogenesis protocols [7–10], such as testicle tissues in two dimensional (2D) [9, 11] or three dimensional (3D) cultures [7, 8, 11], as well as microfluidic platforms [7, 9, 11] reveal limited efficacy due to impaired functional haploid germ cell maturation. Recently, mouse [9, 11, 12] and human [12–14] PSC-derived SSCs [15, 16] have been proposed as an autologous source for azoospermic patients with no or few SSC reserves [15]. However, currently the hiPSC-derived male germ cell number is still limited to a yield of only 2–5% [11, 16]. Thus, novel strategies are required to enhance the yield of autologous iPSCs-derived functional germ cells for clinical translation.

Endocannabinoid system (ECS) components have been found in mESCs [17–20], epiblast like (mEpiLCs) [19] and primordial germ cells (mPGCs) [19]; rodent and human SSC niche [21–23] and human invasive cytotrophoblasts [23–29]. Endogenous AEA and 2-AG gradually increase in human fetal testes with CB1R and CB2R expression on human germ cells, Sertoli and Leydig cells [29, 30]. Exogenous cannabinoid agonists induce embryonic body (EB) formation, reduce apoptosis of mESCs [18] and induce germ cell differentiation in human adult testicular cultures [30]. CB1R and CB2R expression is higher in mature sperm of fertile men compared to infertile males [27] and a gradient of endogenous AEA guides sperm motility in humans [23]. The involvement of ECS in somatic and germline cells of male gonads throughout the developmental stages may indicate a potential guidance for self-renewal and differentiation. Furthermore, CB receptor expression by mESCs that are similar to iPSCs suggests that stimulation of the ECS could be used as a novel strategy to acquire functional, autologous iPSCs-derived hSSCs at sufficient yields for clinical translation.

In this study, we wanted to assess whether hiPSCs reprogrammed from human dermal fibroblasts (hDFs) and hSSCs differentiated from these hiPSCs express ECS components, and if so, whether cannabinoid ligands could induce enrichment of the PSC pool and increased differentiation efficiency towards spermatogenic direction to serve as an autologous cellular therapeutic tool for MFI. To test this hypothesis, we reprogrammed hiPSCs from hDFs using a non-integrating method and characterized them at protein and mRNA levels. Then, we differentiated hSSCs from hiPSCs and characterized the male germ stem cells at protein and mRNA levels. Next, we mapped and quantitatively assessed the distribution profile of endocannabinoid ligands (AEA and 2-AG) and receptors (CB1R, CB2R, TRPV1, and GPR55) in somatic, pluripotent and germ stem cell populations. Finally, we determined the real-time submaximal agonistic concentration to set a therapeutic dose window for the use of CB65 to increase the yield of the germ stem cell pool and translation to male infertility programs.

## Methods

### Experimental design

**Fig. 1:**
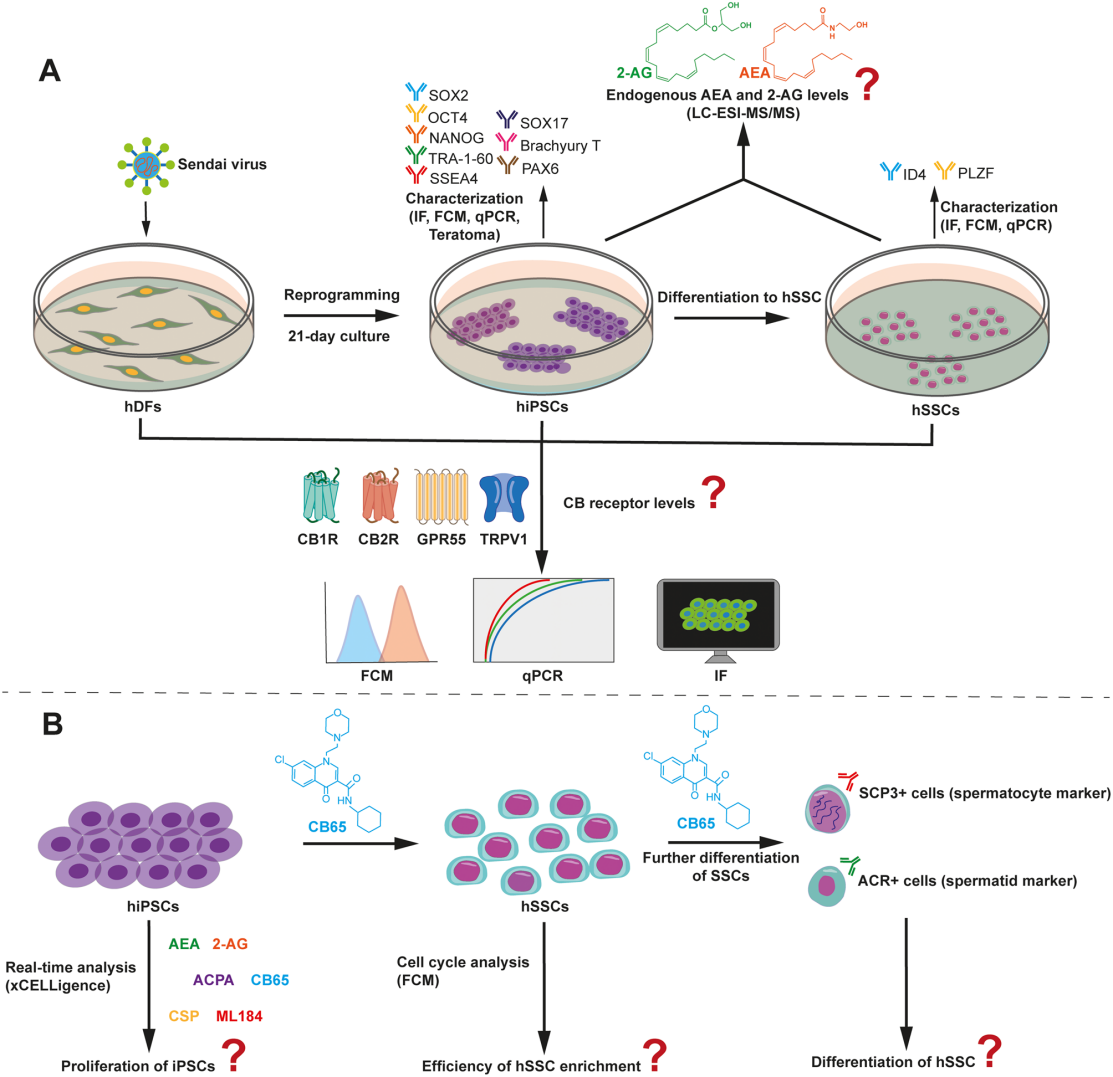
Workflow of the study. **A** Characterization of hiPSCs, hSSCs and the assessment of ECS involvement. **B** CB agonistic efficacy on hiPSCs and hSSCs.

The in vitro study was designed with control and experimental groups, ensuring a 95% accuracy of the output. The dependent variables were characterization and yield parameters, and the independents were time and groups. All work packages were carried out by a minimum of three biological repeats (Fig. 1). The hiPSCs were generated by reprogramming using a commercially available, healthy human adult donor-derived primary fibroblast line (hDF, PCS-201-012, ATCC, USA) [31].

### Reprogramming and characterization of HiPSCs

The hDF cell line reprogrammed at passage 3. The hDFs were cultured in a Dulbecco’s Modified Eagle’s medium- High Glucose (DMEM-HG, BI, USA) with 10% fetal bovine serum (FBS, Pan Biotech., Germany), 1% penicillin-streptomycin (BI, USA) and 1% L-glutamine (BI, USA). The hDFs were reprogrammed into hiPSCs with Yamanaka factors (*OCT4*,* SOX2*,* c-MYC*,* KLF4*) using the CytoTune-iPS 2.0 Sendai Reprogramming Kit (Invitrogen, USA) [31]. Briefly, 28 × 10^4^ hDFs/well were incubated with CytoTune-iPS 2.0 Sendai Reprogramming factors and EmGFP Sendai Fluorescence Reporter (Invitrogen, USA) overnight at 37^o^C. Cells were seeded on hESC-qualified Matrigel (Corning, USA)-coated plates, medium was changed to mTeSR Plus (Stemcell Tech., Canada) and refreshed daily. hiPSC colonies were observed on day 21 and harvested manually or by ReLeSR (Stemcell Tech., Canada) for characterization and further experiments from passage 15 to 20.

The undifferentiated state hiPSCs was quantitively confirmed at the protein level by immunofluorescence labeling (IF), flow cytometry (FCM) and at mRNA level by qPCR analysis [32–34], according to the International Society for Stem Cell Research (ISSCR) guidelines [16, 33, 35–37]. No karyotype analysis was performed. The three-germ layer differentiation capacity of the hiPSCs was evaluated by the teratoma assay in vivo and through induction in vitro. For IF labeling, colonies were seeded on 96-well plates, fixed in 4% paraformaldehyde for 20 min, permeabilizated in 0.2% Triton-X-100 (SERVA, Germany) for 15 min, blocked with 3% goat serum (Abcam, UK), 0.1% Triton-X-100 for 30 min, incubated with primary antibody (Suppl. Table 1) containing 3% bovine serum albumin (BSA, Sigma, Germany) at 4^o^C overnight and with secondary antibody (Suppl. Table 1) in 3% BSA for 1 h at RT. Following nuclear staining by DAPI (Invitrogen, USA) cells were imaged using an Olympus IX73 fluorescence microscope (Olympus, Japan). For qPCR, colonies were incubated with RiboEx™ (GeneAll, Korea) for 5 min, cell homogenates were centrifuged at 12.000 x*g* and the clear phase was incubated with chloroform for 2 min. Cell homogenates were centrifuged with a mini spin column and the solutions present in the kit (GeneAll, Korea). cDNA was synthesized using the WizScript cDNA synthesis kit (WizBio, China). *OCT4*,* SOX2* and *NANOG* pluripotency gene expression was normalized using the *B2M* housekeeping gene (Suppl. Table 2) presented as 2^− dd(Ct)^. For FCM analysis, colonies were detached with ReLeSR, fixed in 4% paraformaldehyde for 15 min at 4^o^C, permeabilized with 0.2% Tween-20 for 20 min, incubated with primary antibodies (Suppl. Table 1) in PBS containing 1% BSA for 1 h at 4 °C; with secondary antibodies (Suppl. Table 1) in PBS containing 0.2% BSA for 30 min at 4^o^C; and analyzed using 20.000 events using the NovoCyte flow cytometer (Acea, USA) and NovoExpress software (Acea, USA). For the teratoma assay, busulfan was injected i.p. as a single dose of 25 mg/kg into Balb/c-Rag2-/- mouse (generously provided by Prof. Dr. Gerard Wagemaker, Erasmus Medical Center, The Netherlands). After 24 h, 10^6^ hiPSCs in Matrigel/DMEMF12 were injected into the right and left *gastrocnemius* muscle of the mice. After four weeks, the macroscopically visible teratomas were removed and fixed with 10% formalin solution. The teratomas were carefully washed with PBS and processed in a vacuum tissue processor (Leica, Germany) using graded alcohols and xylene before being embedded in paraffin. The paraffin blocks were sectioned at 3 μm thickness using a microtome (Leica, Germany). Teratomas were stained with hematoxylin and eosin (H&E) and Masson’s trichrome (MT). The micrographs were obtained using a digital camera (Leica DFC7000 T, Wetzlar, Germany) attached to a light microscope (Leica DMB6 B, Wetzlar, Germany) [32]. For three germ layer differentiation of hiPSCs in vitro, 10^5^ hiPSCs were seeded in a 48-well plate and cultured in mTeSR Plus supplemented with 10 µM ROCKi for 24 h. hiPSCs were washed with DPBS (Gibco, USA) and endodermal, mesodermal and ectodermal differentiation were induced using the STEMdiff™ Trilineage Differentiation Kit (Stemcell Tech., Canada). After differentiation was completed, cells were labeled with primary antibodies designed to detect germ layer-specific markers (Suppl. Table 1) using IF, as described above.

### Differentiation of hiPSC into hSSCs and characterization of hSSCs

hiPSCs were induced to hSSCs when cells reached 80–90% confluency in a Matrigel-coated 12-well plate using a one-step, 10 day long differentiation protocol [16, 37, 38] comprising of MEM-α (BI, USA) supplemented with 3% knockout serum replacement (KSR, Gibco, USCA), 1% insulin-transferrin-sodium selenite (ITS, Sigma, Germany), 0.02% lipid mixture (Sigma, Germany), 1% penicillin-streptomycin, 10 mM HEPES (Sigma, Germany), 2 mM L-glutamine, 60 µM putrescine (Sigma, Germany), 50 µM 2- mercaptoethanol, 1 ng/ml human basic Fibroblast Growth Factor (hbFGF, Peprotech, UK), 20 ng/ml human glial cell line-derived neurotrophic factor (GDNF, Biolegend, USA). IF, FCM, and qPCR were used to characterize the SSCs on day 10 following similar protocols used for the hiPSCs, but using SSC-specific antibodies and primers (Suppl. Tables 1, 2).

### Evaluation of endogenous AEA and 2-AG levels in hiPSCs and hSSCs

Liquid chromatography-electrospray-ionization/tandem mass spectrometry (LC-ESI-MS/MS) (Shimadzu, Japan) was used to analyze the supernatants of hiPSCs and hSSCs according to our previously published protocol [39]. Briefly, supernatants collected within 24 h after refreshing cell medium passed through polymeric sorbent-based solid-phase extraction cartridges that were activated with methanol; then elimination of AEA and 2-AG was performed. Chromatographic separation was conducted by a dynamic phase of acetonitrile-containing formic acid on a C_18_ column (Thermo, USA).

### Assessment of endocannabinoid receptors on hDFs, hiPSCs, and hSSCs

IF, FCM and qPCR were used to evaluate the distribution of CB1R, CB2R, TRPV1 and GPR55 on hDFs, hiPSCs and hSSCs [40]. Briefly, the original hDFs, two different clones of hiPSCs and hSSCs were detached by 0.25% Trypsin, ReLeSR and accutase, respectively. All cells were fixed, permeabilized and labeled using antibodies and primers detailed in Suppl. Table 1, as described above.

### Evaluation of CB2R agonistic effects on proliferation of hiPSCs using RTCA

The impedance-based real-time cell analyzer (RTCA) (xCELLigence, Agilent, USA) was used to evaluate the effective time and dose window of AEA, 2-AG, ACPA, CB65, Capsaicin (CPS), and ML184 on hiPSCs, according to our group’s previously published protocol [40]. Briefly, the biosensor system tracks cell proliferation by collecting the data with (Ω) from the wells of the E-plate (Agilent, USA) according to the changing morphologic behavior of the cells, with intervals of 15 min without interruption for a predetermined period. The software automatically calculates the cell index (CI) and the collected data forms the graph of CI [41, 42]. The hiPSCs were seeded on Matrigel-coated E-Plates at a density of 15 × 10^3^ cells/well [43]. All agonists, control vehicle (0.001% ethanol for AEA, 2-AG, ACPA, CSP and 0.001% DMSO for CB65, ML-184) and control mTeSR Plus medium were applied daily to adherent hiPSCs at a dose range of 10^− 9^- 10^− 5^ M and real-time impedance measurements were performed.

### Evaluation of CB2R agonistic effects on pluripotency of hiPSCs

FCM was used to analyze the presence of pluripotency markers on the hiPSCs following CB65 administration at EC_50_ [32, 40].

### Evaluation of CB2R agonistic effects on differentiation of hiPSC- derived hSSCs

FCM and qPCR were used to assess expression- of stage-specific markers ID4 [44] for hSSCs, PLZF [45] for hSSCs on day 10. ID4 was used as a marker for the A_dark_ to A_pale_ states (early states) of hSCCs and PLZF was chosen as a marker for the A_dark_ to B states (late states) of hSSCs [44]. The meiotic haploid primary/secondary spermatocytes were evaluated using analysis of *SCP3* [46] and round/elongated spermatids using *ACR* [47] on day 12 following CB65 administration of EC_50_ [7, 45].

### Statistical analysis

The data were tested for normality of the distribution by Shapiro-Wilk. We analyzed the parametric data by two-way analysis of variance (ANOVA) and the multiple comparisons were analyzed with the post-hoc Tukey test. Descriptive results were presented as mean ± standard deviation. Analyses were performed and plotted with GraphPad Prism version 9 (GraphPad, USA) with a 95% confidence interval.

## Results

### hiPSCs were reprogrammed from dermal fibroblasts and presented characteristic pluripotency markers

**Fig. 2:**
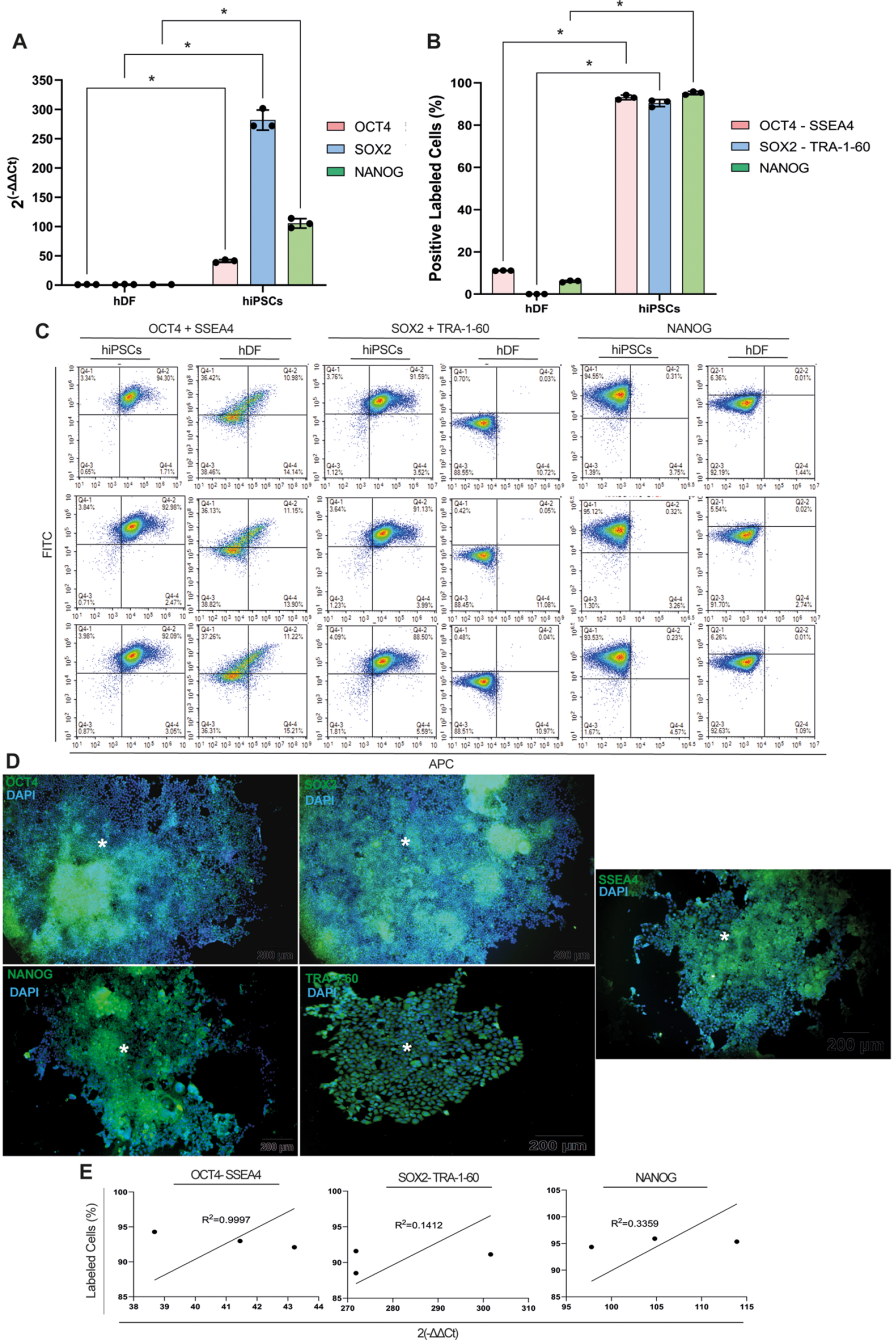
hDFs are reprogrammed to hiPSCs. **A** hiPSCs highly express OCT4, SOX2 and NANOG relative to hDFs. **B.** carry OCT4 + SSEA4, SOX2 + TRA-1-60 and NANOG by FCM. **D** hiPSC colonies (*) present clear and well-defined borders with cytoplasmic OCT4, SOX2, NANOG, SSEA4 and TRA-1-60 immunolabeling by IF. Scale bar is 200 μm, scale bar is 50 μm inlets. **E** Positive correlation for OCT4, SOX2, NANOG, SSEA4 and TRA-1-60 expressions by qPCR and immunolabeling by FCM. * *p* < 0.05

Cuboidal and polymorphic cells formed hiPSC colonies from day 17 (Supp. Figure 1. A- H) and reached the epithelial phenotype on day 19 (Supp. Figure 1. I). The cells expressed a 40-250-fold increase of *OCT4*,* SOX2* and *NANOG* relative to hDFs, confirming the reprogramming of hiPSCs (Fig. 2. A, *p* = 0.0003, *p* < 0.0001 and *p* < 0.0001, respectively). The percentages of OCT4+, SSEA4+, SOX2+, TRA-1-60 + and NANOG + cells in the hiPSC population were significantly increased when compared to that of hDFs by FCM, confirming pluripotency (Fig. 2. B, *p* < 0.0001 for all). Accordingly, the OCT4/SSEA4, SOX2/TRA-1-60 double positive and the NANOG-positive hiPSCs exhibited a range of 92.09–92.98%, 88.50-91.59%, and 93.53–95.12%, respectively (Fig. 2. C). The clearly defined hiPSC colonies displayed a diffuse cytoplasmic immunolabeling for OCT4, SOX2, NANOG, SSEA4 and TRA-1-60 by IF (Fig. 2. D). The stemness marker expression profile at the mRNA level presented a positive correlation with immunolabeling data at the protein level for OCT4/SSEA4 (R^2^ = 0.9997, *p* = 0.0103), SOX2/TRA-1-60 (R^2^ = 0.1412, *p* = 0.7548) and NANOG (R^2^ = 0.3359, *p* = 0.6065), respectively (Fig. 2. E). The injected hiPSCs all formed teratomas, containing cells from endodermal, mesodermal and ectodermal origin in all mice (Supp. Figure 2. A, B) and showed a similar differentiation potential in the in vitro differentiation assay. In teratomas, endodermal lineage-derived liver hepatic lobular parenchyme (Supp. Figure 2. C- E); mesodermal lineage-derived striated muscle, vessel, adipose and connective tissues (Supp. Figure 2. G- I); and ectodermal lineage-derived peripheral nerve formation (Supp. Figure 2. K- M) was observed. IF analysis of in vitro differentiation assays showed that ts differentiated into SOX17 + endodermal cells (Supp. Figure 2. F), Brachyury T + mesodermal cells (Supp. Figure 2. J) and PAX6 + ectodermal cells (Supp. Figure 2. N). Thus, we confirmed pluripotency of the reprogrammed somatic cells.

### hiPSC differentiated to hSSCs expressing characteristic ID4 and PLZF at high levels

**Fig. 3:**
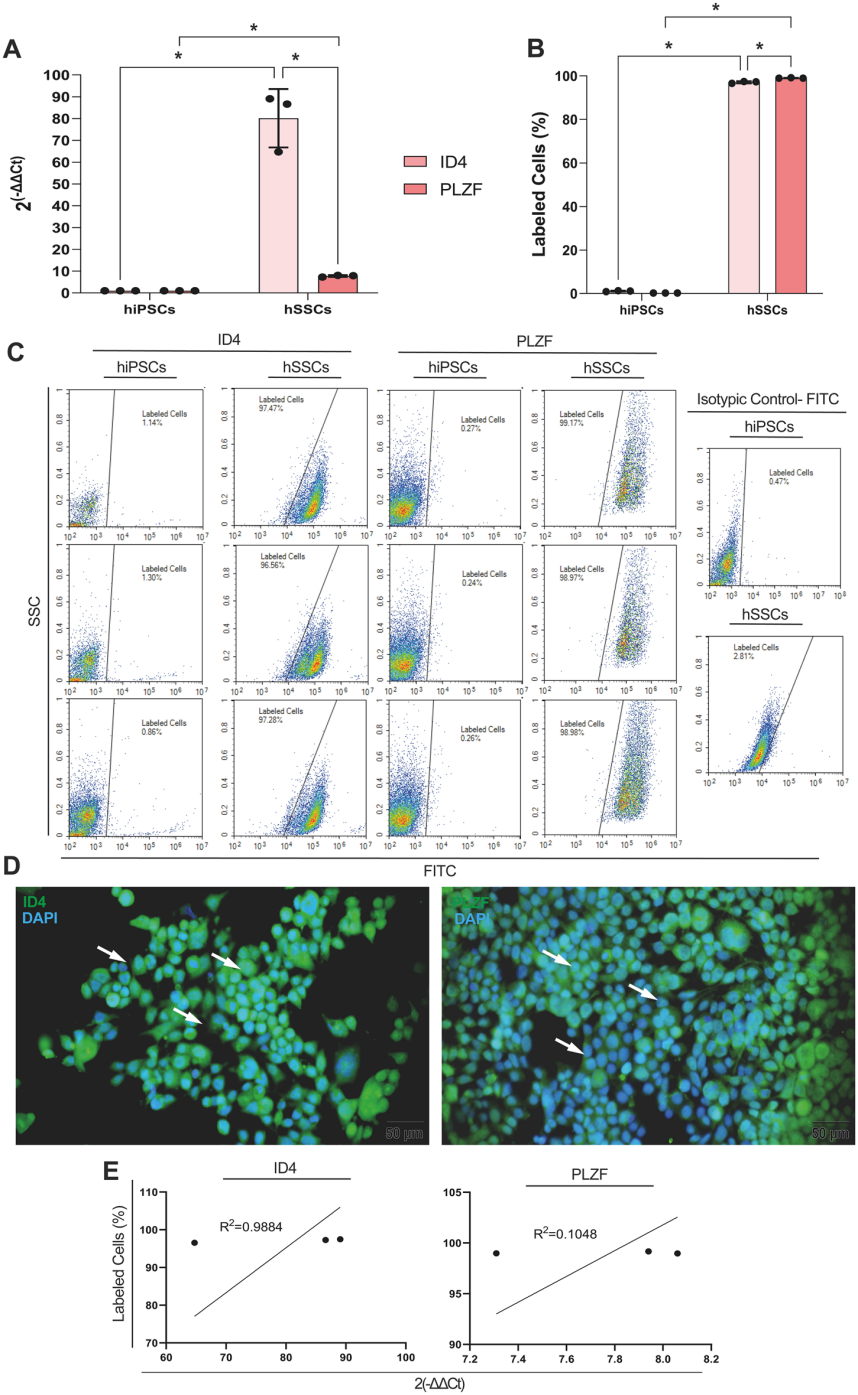
hSSCs are differentiated from hiPSCs. **A** hSSCs highly express ID4 and PLZF relative to hiPSCs. **B**,**C** carries ID4 and PLZF by FCM. **D** ID4 and PLZF label the cytoplasm by IF. Scale bar is 50 μm. **E** Positive correlation for ID4 and PLZF expressions by qPCR and immunolabeling by FCM. * *p* < 0.05

The hSSCs expressed *ID4* and *PLZF* at high levels, presenting 80- and 10-fold changes relative to hiPSCs (Fig. 3. A, *p* < 0.0001 for all). ID4 + and PLZF + hSSCs ranged from 96.56 to 97.47% and 98.97–99.17%, while hiPSCs ranged from 0.86 to 1.30% and 0.24–0.27%, respectively, by FCM (Fig. 3. C). The percentage of ID4 + and PLZF + hSSCs was significantly higher than that of hiPSCs (Fig. 3. B, *p* < 0.0001 for all). ID4 and PLZF presented a homogeneous diffuse cytoplasmic and perinuclear immunolabeling within the hSSCs by IF (Fig. 3. D). The characteristic spermatogonial marker expression profile at mRNA level presented a positive correlation with the immunolabeling data at a protein level for ID4 (R^2^ = 0.9884, *p* = 0.0688) and PLZF (R^2^ = 0.1048, *p* = 0.7901), respectively (Fig. 3. E), confirming differentiation to hSSCs.

### hiPSCs and hSSCs secreted endocannabinoid ligands AEA and 2-AG

**Fig. 4:**
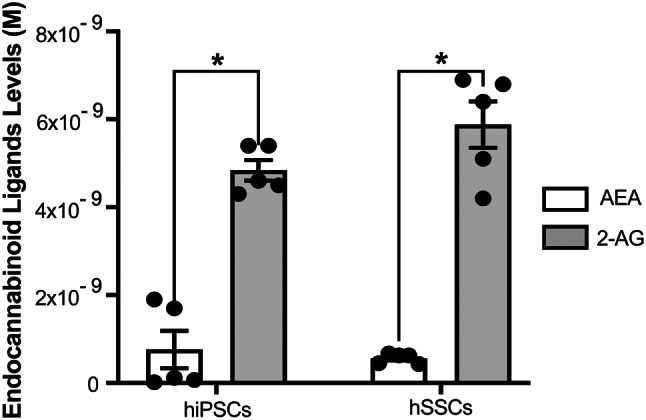
hiPSCs and hSSCs secrete endocannabinoid ligands AEA and 2-AG at pM to nM levels by LC-MS/MS. *p < 0.05

Both hiPSCs and hSSCs secreted AEA and 2-AG to culture supernatants at picomolar (pM) to nanomolar (nM) levels by LC/MS at a similar concentration range (Fig. 4, Suppl. Table 3). The secretion of 2-AG was significantly higher than that of AEA in both hiPSCs and hSSCs (Fig. 4. A, *p* < 0.0001 for all).

### hiPSCs expressed CB1, CB2, TRPV1 and GPR55 receptors

**Fig. 5:**
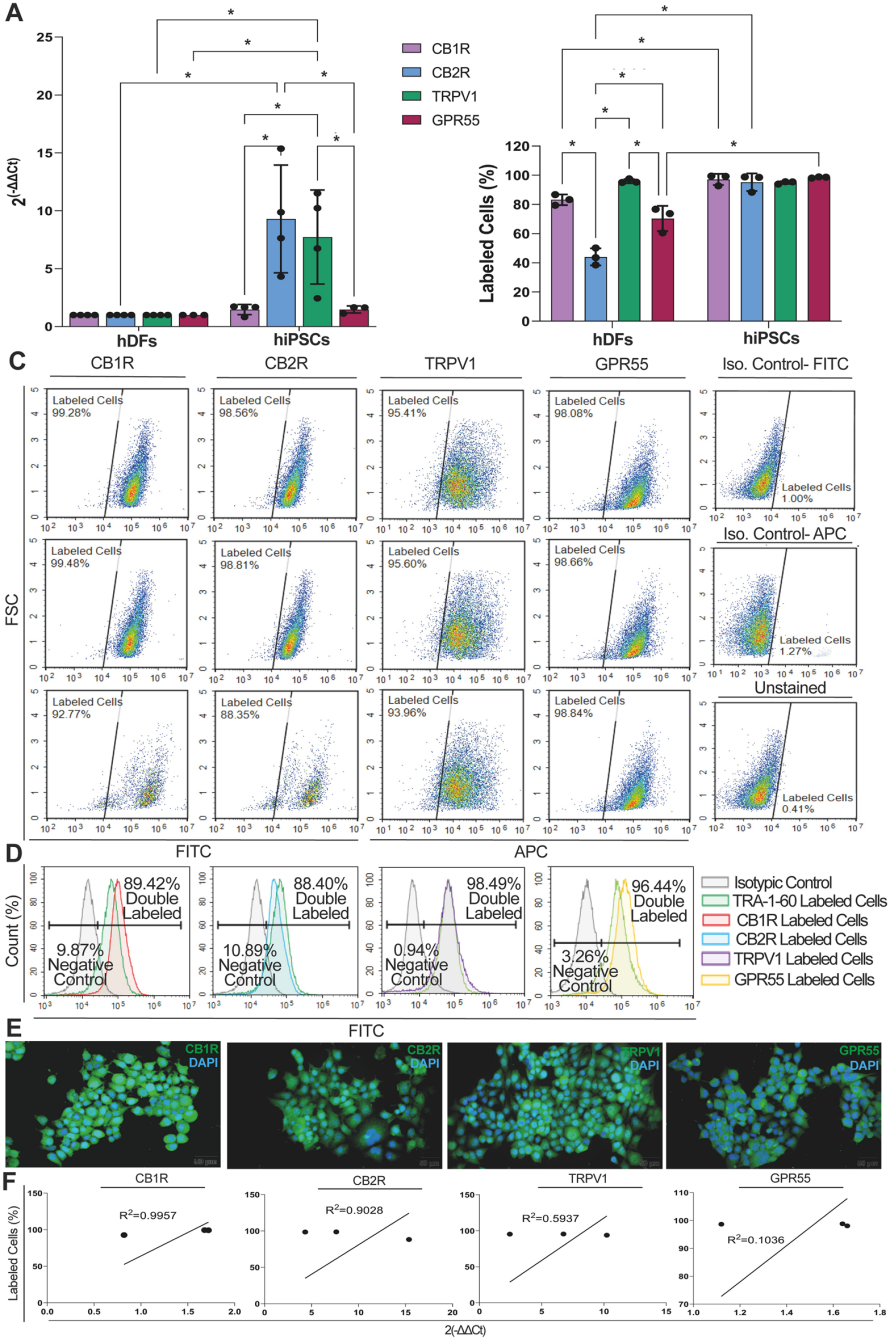
hiPSCs carry cannabinoid receptors. **A** hiPSCs express CB1R, CB2R, TRPV1 and GPR55 relative to hDF. **B** Homogenous high percentage of CB1R, CB2R, TRPV1 and GPR55 distribution on hiPSCs by FCM. **C** CB1R, CB2R, TRPV1 and GPR55 immune distribution in hiPSCs by FCM. **D** Representative diagram for co-distribution of CB1R, CB2R, TRPV1 and GPR55 and stemness marker TRA-1-60 on hiPSCs by FCM. **E** Nucleo-cytoplasmic (*arrow*) labeling of CB1R, CB2R, TRPV1 and GPR55 on hiPSCs by IF. Scale bar is 50 μm. **F** Positive correlation for CB1R, CB2R, TRPV1 and GPR55 mRNA expression by qPCR and immunolabeling by FCM. *p < 0.05

Both hDFs and hiPSCs expressed cannabinoid receptors (*CB1R*,* CB2R*,* TRPV1*, and *GPR55*) at various levels (Fig. 5. A- F, Supp. Figure 3A-C, Suppl. Figure 4^4^, Supp. Table 4, Suppl. Figure 5). The hiPSCs expressed *CB1R*,* CB2R* (*p* = 0.0011), *TRPV1* (*p* = 0.0112), and *GPR55* 1.5, 9.3, 7.7 and 2-fold higher when compared to hDFs, respectively (Fig. 5. A). *CB2R* expression was significantly higher in hiPSCs when compared to *CB1R*,* TRPV1* and *GPR55* (Fig. 5. A, *p* = 0.0022, *p* = 0.0052). IF and FCM confirmed a high level of cannabinoid receptor distribution of CB1R (92.77–99.48%), CB2R (88.35–98.81%), TRPV1 (93.96–95.60%) and GPR55 (98.08–98.84%) in hiPSCs presenting a similar ratio (Fig. 5. B, C, E). FCM also revealed co-expression of pluripotency marker TRA-1-60 and the cannabinoid receptors. The hiPSCs expressed CB1R, CB2R, TRPV1 and GPR55 together with TRA-1-60 at 89.42%, 88.40%, 98.49% and 96.44%, respectively (Fig. 5. D). The cannabinoid receptors expression pattern presented a highly positive correlation with immunolabeling data by FCM (Fig. 5. F, R^2^ = 0.9957 and *p* = 0.0417, R^2^ = 0.9028 and *p* = 0.2019, R^2^ = 0.5937 and *p* = 0.4400, R^2^ = 0.1036 and *p* = 0.7914, respectively).

### hSSCs expressed high levels of CB2R and low levels of CB1R, TRPV1, GPR55

**Fig. 6:**
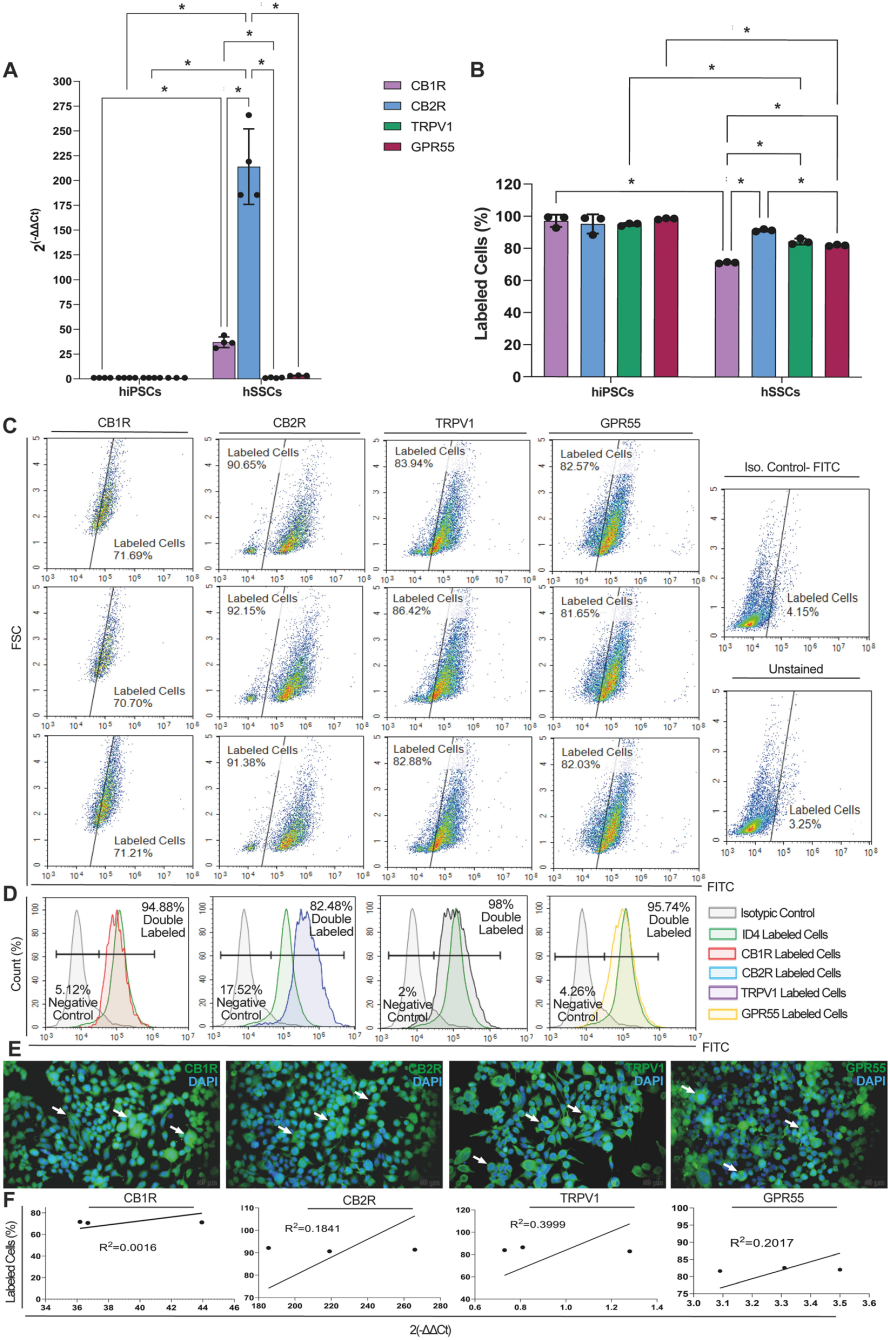
hSSCs carry cannabinoid receptors. **A** hSSCs express high CB2R and low CB1R, TRPV1 and GPR55 relative to hiPSCs. **B** CB2R presents a significantly higher ratio compared to CB1R, TRPV1 and GPR55 on hSSCs. **C** CB1R, CB2R, TRPV1 and GPR55 immune distribution in hiPSCs by FCM. **D** Representative diagram for co-distribution of CB1R, CB2R, TRPV1 and GPR55 and SSC marker ID4 on hSSCs by FCM. **E** Cytoplasmic (*arrow*) labeling of CB1R, CB2R, TRPV1 and GPR55 on hSSCs by IF. Scale bar is 50 μm. **F** Positive correlation for CB1R, CB2R, TRPV1 and GPR55 mRNA expression by qPCR and immunolabeling by FCM. * *p* < 0.05

hSSCs expressed all cannabinoid receptors (Fig. 6, Suppl. Table 4, Suppl. Figure  5). hSSCs carried CB1R, CB2R and TRPV1 at ranges of 70.70-71.69%, 90.65–92.15%, 82.88–86.42%, and 81.03–82.57% by FCM, respectively (Fig. 6. B, C). *CB2R* expression was significantly higher when compared to *CB1R*,* TRPV1* and *GPR55* (Fig. 6. A, *p* < 0.0001 for all). Additionally, *CB2R* expression by hSSCs was 200-fold higher than that of hiPSCs (Fig. 6. A, *p* < 0.0001). Similarly, numbers of CB2R positive hSSCs were significantly higher than CB1R, TRPV1 and GPR55 positive hSSCs by FCM (Fig. 6. B, C, *p* < 0.0001, *p* = 0.0002 and *p* < 0.0001, respectively). The hSSCs expressed CB1R, CB2R, TRPV1 and GPR55 together with ID4 at 94.88%, 82.48%, 98% and 95.74%, respectively (Fig. 6. D). Gene expression data of hSSCs demonstrated a positive correlation with FCM for CB1R (R^2^ = 0.001613, *p* = 0.9744), CB2R (R^2^ = 0.3999, *p* = 0.7176), TRPV1 (R^2^ = 0.1841, *p* = 0.5642) and GPR55 (R^2^ = 0.2017, *p* = 0.7035).

### CB2R agonism induced proliferation of hiPSCs

**Fig. 7:**
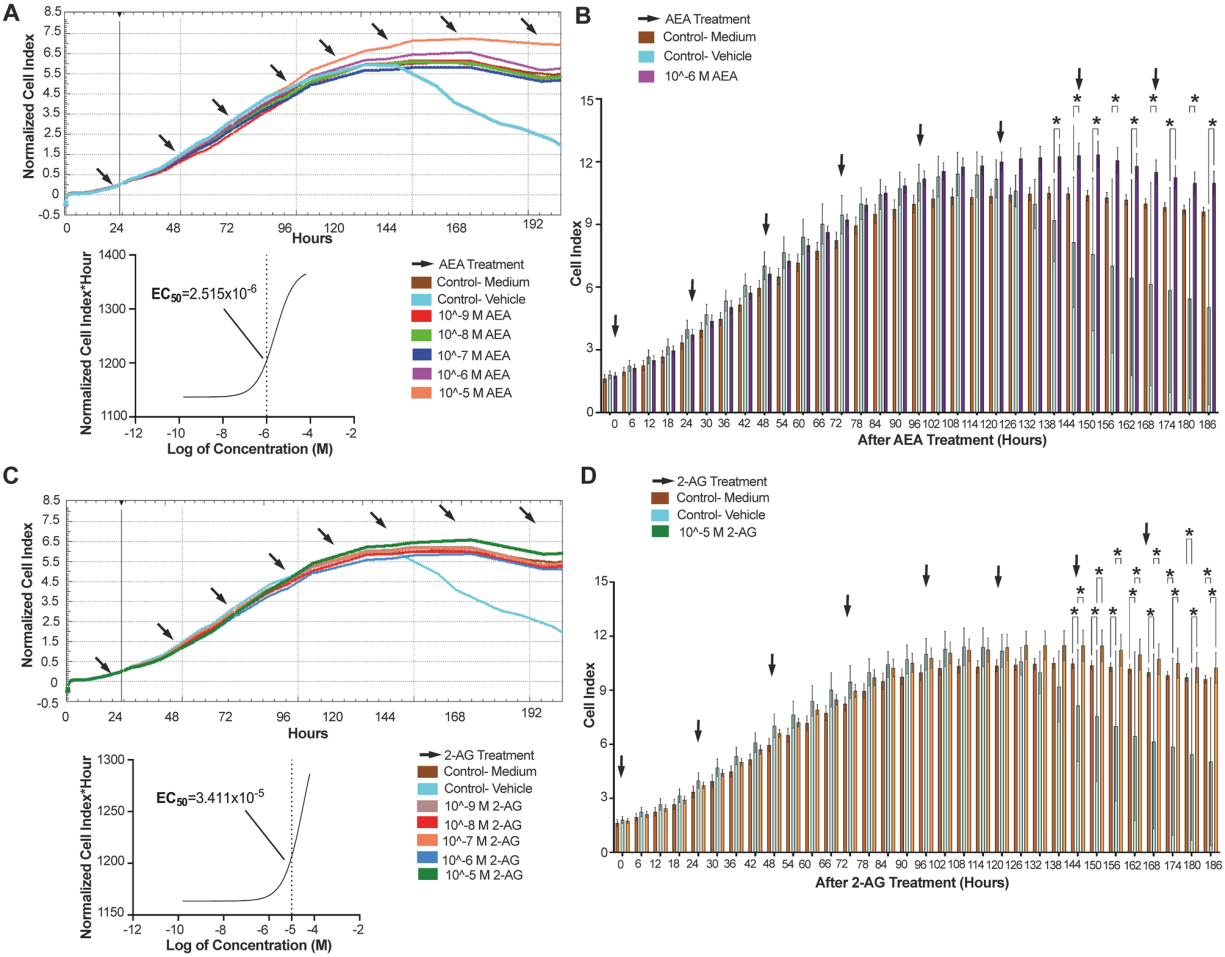
AEA and 2-AG slightly induce the proliferation of hiPSCs. **A**. Normalized CI values of 10^− 9^ to 10^− 5^ M AEA- applied groups in comparison to medium and vehicle controls (%0.001 ethanol) for 200 h sorting the EC_50_ for AEA by RTCA. **(B)** AEA presents a therapeutic proliferative window at 10^− 6^ M between 138–186 h. **(C)** Normalized CI of 10^− 9^- 10^− 5^ M 2-AG applied groups in comparison to medium and vehicle controls for 200 h sorting the EC_50_ for 2-AG by RTCA. **(D)** 2-AG presents a therapeutic proliferative window at 10^− 5^ M from 144 to 186 h. * *p* < 0.05

**Fig. 8:**
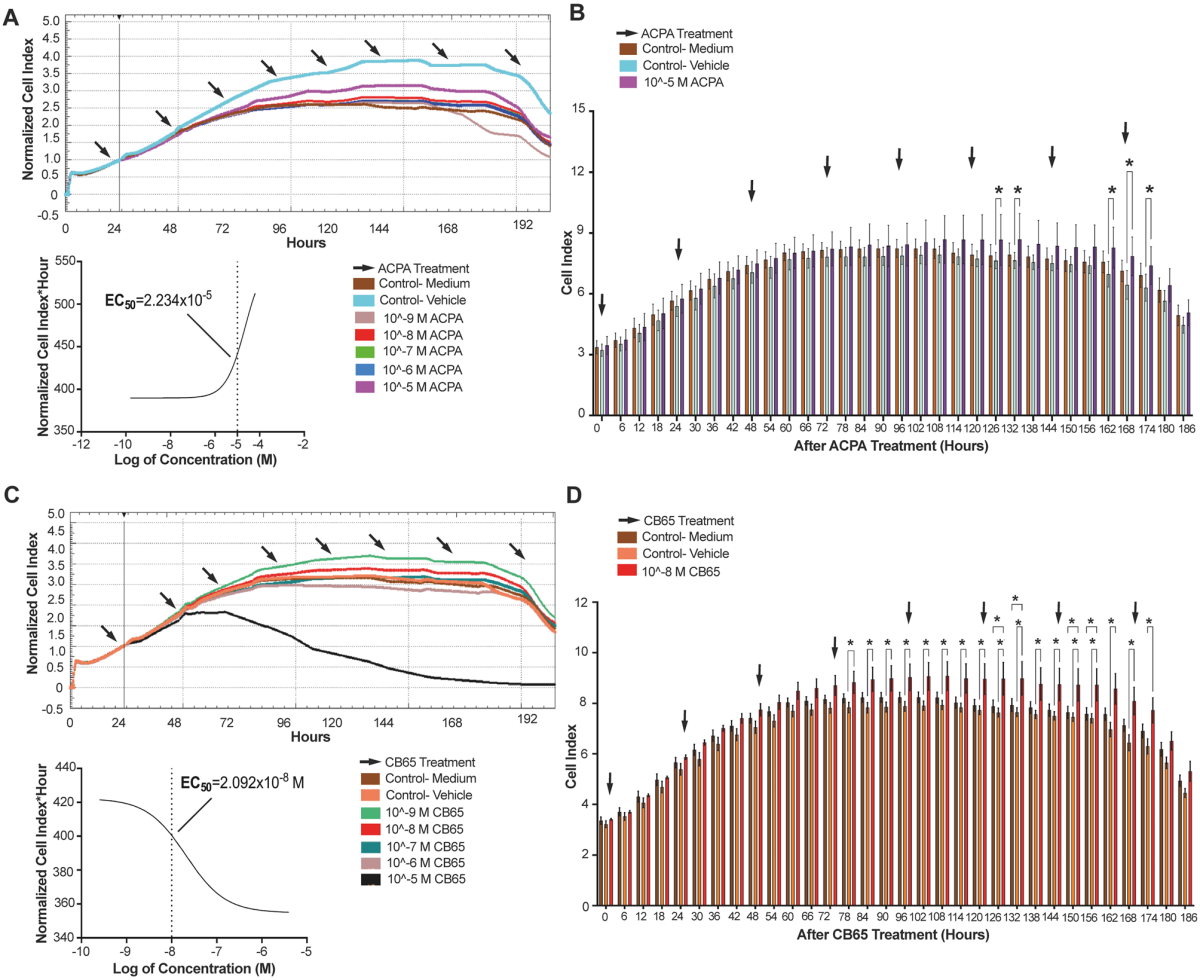
CB65 increases the proliferation of hiPSCs, while ACPA reduces the proliferation **(A)** Normalized CI values of 10^− 9^- 10^− 5^ M ACPA- applied groups on hiPSCs for 200 h in comparison to medium and vehicle controls (%0.001 ethanol) applied controls sorting EC_50_ for ACPA by RTCA. **(B)** ACPA presents a therapeutic window at 10^− 5^ M on 126, 132 and 162–174 h. **(C)** Normalized CI values of 10^− 9^- 10^− 5^ M CB65 applied groups for 200 h in comparison to medium and vehicle applied (%0.001 DMSO) controls, sorting the EC_50_ value for CB65 by RTCA. **(D)** CB65 presents a therapeutic proliferative window at 10^− 8^ M between 78 and 174 h. * *p* < 0.05

**Fig. 9:**
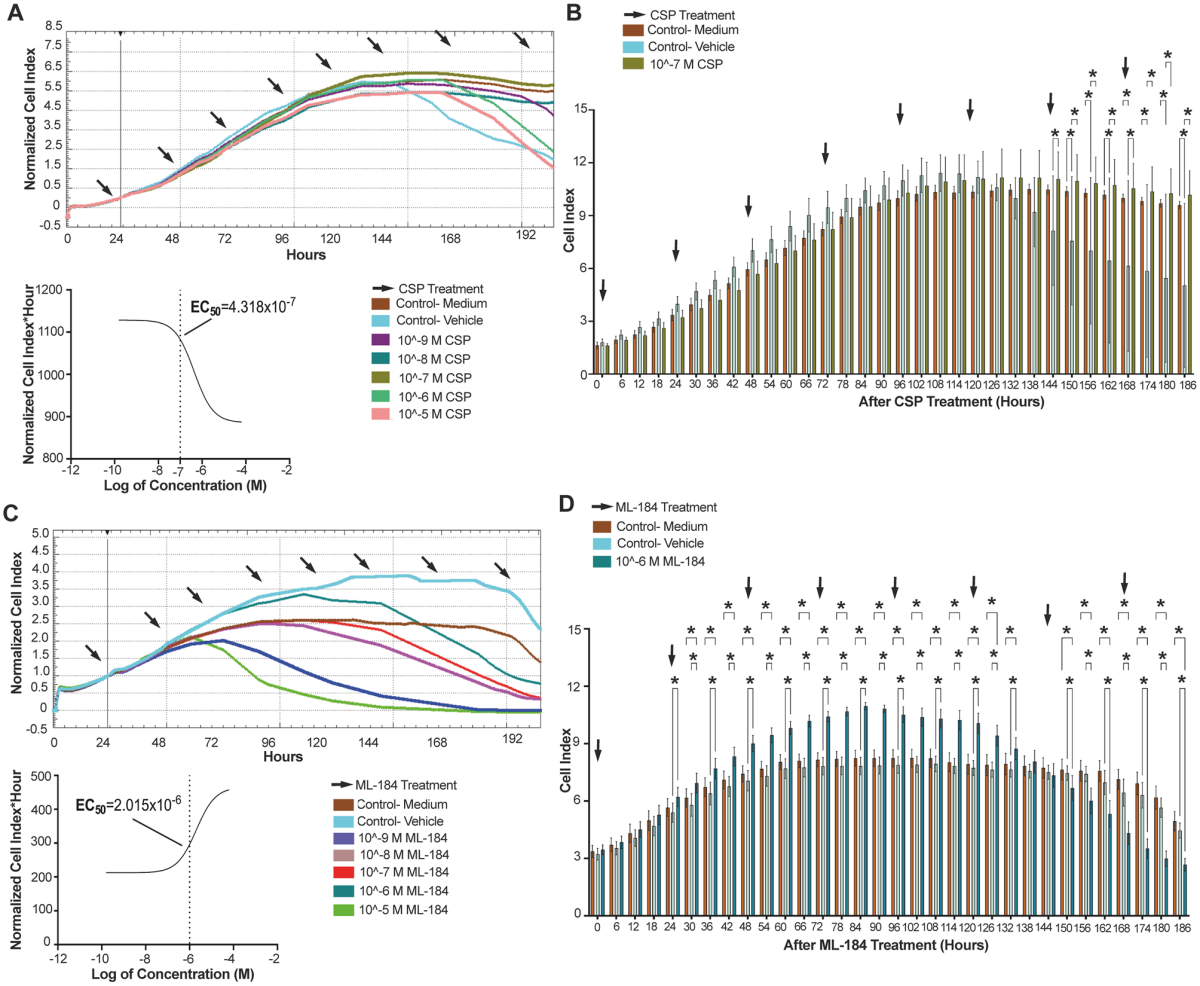
CSP slightly increases proliferation of hiPSCs, while ML184 decreases the proliferation. **(A)** Normalized CI values of 10^− 9^- 10^− 5^ M CSP- applied groups for 200 h in comparison to medium and vehicle controls (%0.001 ethanol) sorting the EC_50_ for CSP by RTCA. **(B)** CSP presents a therapeutic proliferative window at 10^− 7^ M between 144 and 186 h. **(C)** Normalized CI values of 10^− 9^- 10^− 5^ M ML-184 applied groups for 200 h in comparison to medium and vehicle (%0.001 DMSO) applied controls sorting the EC_50_ for ML-184 by RTCA. **(D)** ML-184 presents a therapeutic window at 10^− 6^ M between 24–132 and 150–186 h. * *p* < 0.05

The CB1R endogenous agonist AEA induced proliferation of hiPSCs at an EC_50_ value of 2.515 × 10^− 6^ M within the time window of 138 to 186 h following administration (Fig. 7. A, B). However, the induction was not statistically significant compared to medium only and vehicle controls (Fig. 7. A, B). The CB2R endogenous agonist 2-AG induced proliferation of hiPSCs at an EC_50_ value of 3.411 × 10^− 5^ M within the time window of 144 to 186 h following administration but was not statistically different from medium only or vehicle controls (Fig. 7. C, D). The CB1R selective synthetic agonist ACPA decreased the proliferation rate of hiPSCs at an EC_50_ value of 2.234 × 10^− 5^ M within the time window from 126 to 174 h following administration but this was not significantly different from vehicle controls (Fig. 8. A, B). The CB2R selective synthetic agonist CB65 increased the proliferation rate of hiPSCs with an EC_50_ value of 2.092 × 10^− 8^ M between the time window of 78 to 174 h following administration and was statistically significant when compared to both medium and vehicle controls (Fig. 8. C, D). The TRPV1 selective synthetic agonist CSP induced proliferation of hiPSCs at an EC_50_ value of 4.318 × 10^− 7^ M within the time window of 144 to 186 h following administration and was not significantly different from medium and vehicle controls (Fig. 9. A, B). The GPR55 selective synthetic agonist ML184 decreased the proliferation rate of hiPSCs at an EC_50_ value as 2.015 × 10^− 6^ M within the time window of 24 to 186 h following administration and was not significantly different from controls (Fig. 9. C, D). The selective synthetic agonist of AEA and CB1R, ACPA exhibited opposite effects on proliferation of hiPSCs, suggesting possible induction via CB2R agonism by both AEA and 2-AG. Consequently, the CB2R selective synthetic agonist CB65 which has a higher affinity to CB2R when compared to 2-AG, demonstrated stimulation of proliferation at a lower submaximal dose and was able to significantly increase the proliferation rate of hiPSCs at 10^− 8^ M from 78 h (Fig. 8. C, D).

### CB2R agonism maintained pluripotency of hiPSCs

**Fig. 10:**
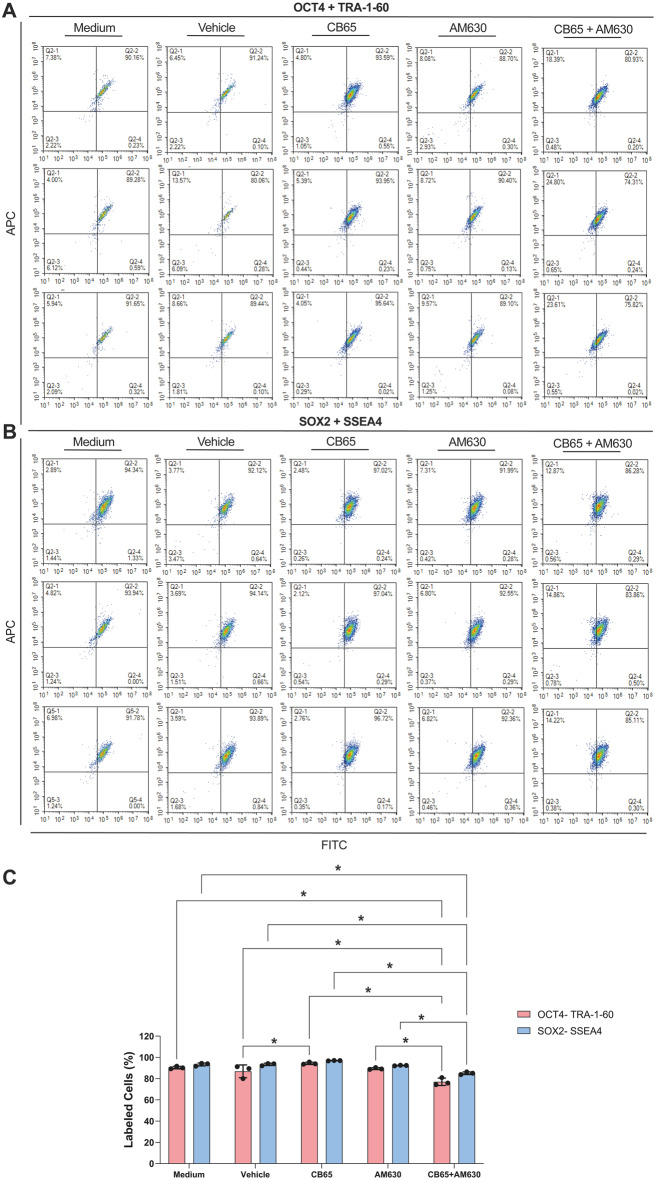
CB65 maintains the pluripotency of hiPSCs. CB65 slightly increases **(A)** OCT4- TRA-1-60 and **(B)** SOX2-SSEA4 double immune distribution of hiPSCs by FCM. **(C)** OCT4- TRA-1-60 presents a higher ratio in CB65 applied hiPSCs groups compared to vehicle (%0.001 DMSO) and CB65 + AM630 applied groups. * *p* < 0.05

The CB2R synthetic receptor agonist CB65 further increased the percentage of OCT4/TRA-1-60 double positive hiPSCs compared to medium, vehicle and CB65 + AM630 treated groups at 78 h following daily application at a submaximal dose (2.092 × 10^− 8^ M, Fig. 10. C, *p* = 0.0096, *p* < 0.0001). Subsequent application of an antagonist blocked the agonistic effect, confirmed by the appearance of similar patterns to the medium vehicle and CB65 + AM630 treated groups. CB65 also increased the percentage of SOX2/SSEA4 double positive hiPSCs compared to CB65 + AM630 treated groups at 78 h following daily application at a submaximal dose (2.092 × 10^− 8^ M, Fig. 10. C, *p* = 0.0037). Again, application of an antagonist blocked the agonistic effect, as confirmed by the presence of similar growth patterns of medium, medium vehicle and the CB65 + AM630 treated groups. OCT4/TRA-1-60 double positive cells constituted 89.28–91.65% and 80.06–91.24%, 93.59–95.64%, 88.70–90.40%, and 74.31–80.93% of medium and vehicle controls, CB65, AM630 and CB65 + AM630 groups at 78 h (Fig. 10. A). SOX2/SSEA4 double positive cells made 91.78–94.34%, 92.12–94.14%, 96.72–97.04%, 91.99–92.55% and 83.86–86.28% of medium and vehicle controls, CB65, AM630 and CB65 + AM630 treated groups (Fig. 10. B). Thus, CB65 maintained the pluripotency of hiPSCs, as confirmed by co-expression of pluripotency markers following daily application at a submaximal concentration.

### CB2R agonism induced the yield of proliferative state hSSCs

**Fig. 11:**
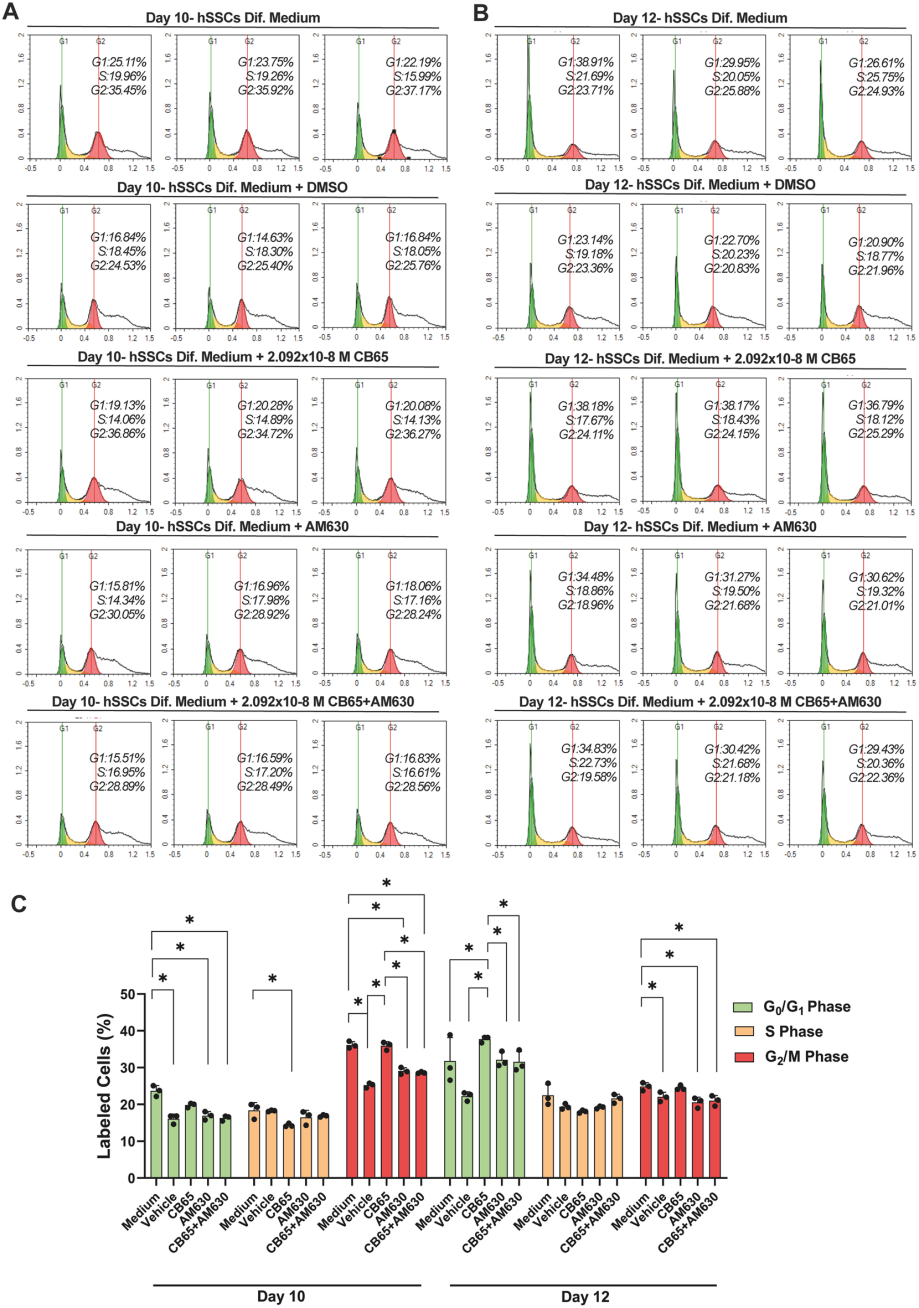
CB65 induces proliferation of hSSCs. CB65 increases the percentage of hSSCs in **A**. G_2_/M phases on day 10 and **(B)** G_0_/G_1_ phases on day 12 by PI labeling. **(C)** CB65 at submaximal dose increases the percentage of hSSCs in G_2_/M and G_0_/G_1_ phases on day 10 and 12, respectively, compared to medium, medium vehicle (%0.001 DMSO), AM630 and CB65 + AM630 applied groups. *p < 0.0001

CB65 at submaximal dose also increased the percentage of hSSCs in G_2_/M and G_0_/G_1_ phases on day 10 (Fig. 11. A) and 12 (Fig. 11. B), respectively, compared to medium, medium vehicle, AM630 and CB65 + AM630 treated groups (*p* < 0.0001 for all multiple comparisons, Fig. 11). In contrast, the CB2R synthetic receptor antagonist AM630 blocked the agonistic effects of CB65 and decreased the ratio of hSSCs in G_0_/G_1_, S, and G_2_/M phases compared to medium control and CB65 applied groups on day 10 and 12 (*p* < 0.0001 for all multiple comparisons, Fig. 11. C). Therefore, it is likely that CB65 increased hSSC proliferation by promoting entrance of hSSCs to the cell cycle.

### CB2R agonism induced differentiation of hSSCs

**Fig. 12:**
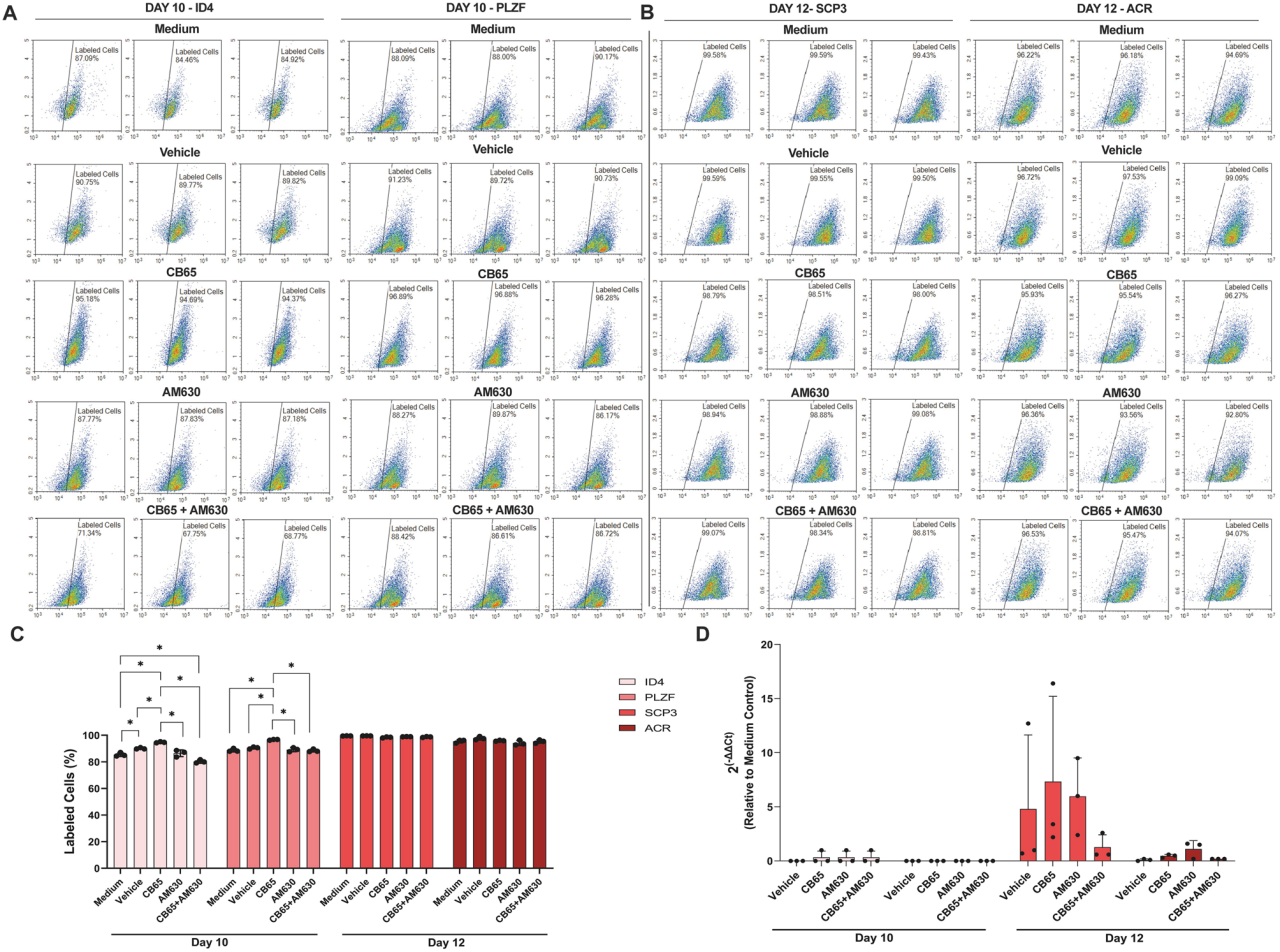
CB65 induces differentiation of hSSCs. CB65 at submaximal dose **A**,** C.** increases the ratio of ID4+, PLZF + hSSCs on day 10 and **B**,** C.** does not influence the ratio of SCP3+, ACR + hSSCs on day 12 compared to controls by FCM. **D.** On day 10, all groups present a similar ID4 and PLZF expression pattern compared to controls. On day 12, CB65 applied group presents a higher SCP3 and a similar ACR expression compared to controls. * *p* < 0.05

CB65 induced an increase in ID4 + and PLZF + hSSCs compared to medium, vehicle, AM630, and CB65 + AM630 treated groups on day 10 when applied at a submaximal dose (Fig. 12. A, C, *p* = 0.0003 for CB65- vehicle control comparison, *p* < 0.001 for other all multiple comparisons). AM630 blocked the effect of CB65 and decreased ID4 + and PLZF + hSSCs (Fig. 12. A, C, *p* < 0.001 for all multiple comparisons). CB65 increased *SCP3* expressing haploid spermatocytes compared to vehicle, AM630 and CB65 + AM630 treated groups on day 12 when applied at a submaximal dose (Fig. 12. B- D). CB65 increased the number of *SCP3* expressing haploid spermatocytes compared to vehicle, AM630 and CB65 + AM630 treated groups on day 12 when applied at a submaximal dose (Fig. 12.D). AM630 and CB65 + AM630 treated reduced *SCP3* expressing haploid spermatocytes and *ACR* expressing spermatids on day 12 (Fig. 12. D). CB65 did not affect *ACR* expression and labeling pattern of hSSCs compared to medium, vehicle, AM630 and CB65 + AM630 treated groups on day 12 (Fig. 12. D).

## Discussion

This study implicates for the first time the involvement of ECS in both hDF-derived hiPSCs the and hiPSC-derived hSSCs at an mRNA and protein level. Both hiPSCs and hSSCs secrete endocannabinoid ligands (AEA and 2-AG) and express cannabinoid receptors (*CB1R*,* CB2R*,* TRPV1*, and *GPR55*) at significant levels. 2-AG secretion and the CB2R expression are higher in both hiPSCs and hSSCs when compared to that of AEA and other cannabinoid receptors. Overall, cannabinoid receptor content increased during SSC differentiation. CB65 increases the proliferation of hiPSCs with an EC_50_ value of 2.092 × 10^− 8^ M between 78 and 174 h and supports differentiation and maturation of hSSCs on day 10 and 12, when applied daily compared to control and the CB2R antagonist treated groups.

First, hiPSCs were successfully reprogrammed from hDFs using a non-integrating method and colonies appeared from day 19. Pluripotency was confirmed by NANOG, OCT4, SOX2, SSEA4, and TRA-1-60 expression at the mRNA and protein level. We preferred not to use lentiviral vectors since they might cause genetic modifications due to possible viral genome integration into the genome of recipient patients [48]. Our iPSC reprogramming efficiency using transfer of the *OCT3/4*,* SOX2*,* KLF4*,* c-MYC*,* NANOG* and *LIN28* transcription factors using SeV is consistent with the reprogramming frequency and time frame (typically 16–36 days), as previously described for this method [49–51]. Our protocol provided a sufficient yield of pluripotent stem cell colonies expressing a high percentage of *OCT4*,* SOX2*,* NANOG*,* TRA-1-60* and *SSEA4* in line with the prior works [16, 35–37, 52, 53]. Although karyotyping is recommended by the ISSCR to monitor culture-acquired genetic variations, this is not obligatory and only required for translational studies [53]. Although our hiPSCs were reprogrammed from a commercially available dermal fibroblast cell line derived from a single, healthy donor, this has been shown to be sufficient and this method is commonly used in preliminary studies [33].

Our one-step, xenogen-free SSC-induction protocol is based on critical niche factors (GDNF, bFGF, ITS, putrescine, lipid mixture, and β-mercaptoethanol) and was used to successfully differentiate hiPSCs into ID4+, PLZF + hSSCs reaching a yield of 96% on day 10. Several research groups have previously differentiated adult healthy individual-derived [16, 37, 38, 54] and NOA patients-derived [16, 37] foreskin and dermal fibroblasts into ID4+, PLZF+, GFRa1+, GPR125+, CD90+, PIWIL4+, DAZL+, DMRT1+, UTF1+, VASA + and DAZL + SSCs in 6 days to 4 weeks using one or multi-step chemically induced protocols. Here, we report a novel xeno-free one-step chemical-based protocol by combining the protocols of Wang et al. [37], Zhao et al. [16] and Easley et al. [38] reaching an overall higher yield of hSSCs at 10 days. Our novel differentiation medium demonstrated to be a highly efficient and quick protocol that supported the formation of SSCs with high yield. Our protocol provided between 96.56 and 97.47% and 98.97–99.17 positivity for ID4 and PLZF, respectively. We did not use any genetic modifications or xenogeneic-derived factors out of consideration of clinical translation [48]. Three research groups reported the differentiation of hiPSCs to hSSC using animal cell-hiPSCs co-culture platforms or genetic manipulation with a 75% yield on day 80 [13], a 30% yield on day 60 [55] or no yield at 60 days [56]. Those experimental settings are not applicable to ART protocols due to ethical restrictions [57, 58]. Furthermore, the yield of hSSC derivation ranged from 30 to 78% and the obtained haploid cells remained below ART standards. In addition, these experimental settings are not applicable to ART protocols due to ethical restrictions [58].

Three groups supplemented the one-step differentiation media with insulin, BSA, vitamin C (VC) and/or valproic acid (VPA), providing 78%, 70%, and 60% of hSSC yields, respectively [16, 37, 38]. We replaced BSA with KSR and VC with b-mercaptoethanol and excluded VPA and insulin in our protocol, resulting in a final yield that exceeds previous studies [16, 37, 38]. Xu et al. reported a three-step protocol composed of consecutive temperature inductions with different supplements each, at 37^o^C for 6 days, 34^o^C for 4 days and 34^o^C for 30 days. The first differentiation medium was largely similar to ours but contained insulin and BSA instead of KSR [54]. The second round of differentiation comprised induction in DMEM/F12 medium supplemented with FBS, GDNF, retinoic acid (RA) and stem cell factor (SCF), and in the third stage the cultures were supplemented with GDNF, testosterone, VC, vitamin A (VA), vitamin E (VE) and recombinant human follicle-stimulating hormone (rhFSH) [54]. The three-step protocol reached a yield of up to 80% by the propagation of hSSCs until passage 3 [54]. Our results are limited to 10 days of culture. However, they confirm a reliable differentiation of hSSCs with the presence of common stage-specific markers ID4 + and PLZF + at high yields, in line with the three-step method [16, 37, 38]. Here, we report a xeno-free one-step chemically defined protocol to differentiate hiPSC derived from healthy human fibroblasts into hSSC in 10 days with a 96% yield.

This work further demonstrates for the first time the secretion of the endocannabinoids AEA and 2-AG from hiPSCs and hiPSC-derived hSSCs at pM to nM levels. Differentiation to hSSCs did not significantly change endocannabinoid levels. Mouse ESCs and embryonic fibroblasts secrete AEA and 2-AG at roughly similar pM levels (2.5 × 10^− 14^ M, 3.5 × 10^− 11^ M in ESCs, 6.5 × 10^− 11^ M in MEF) [17]. The AEA secretion of the hiPSCs is ten times, and the 2-AG secretion is a hundred times higher compared to the mESCs in this study. The endocannabinoid secretome is not reported in hESCs in literature. However, our group previously reported AEA and 2-AG secretion by bone marrow-derived mesenchymal stem cells [39] and had similar levels to those of hiPSCs. Taken together, the present study reflects a similar stable endocannabinoid secretome profile for human pluripotent and multipotent stem cells. Adult mouse epididymal sperms secrete 10^− 5^ M of 2-AG, but not AEA [59]. Previous studies report AEA secretion from the ejaculation of fertile and infertile human males in a similar range between 10^− 7^ to 10^− 11^ M [17, 39, 59]. However, 2-AG secretion was found to range from 10^− 11^ to 10^− 8^ M and differed depending on fertility, with ten to hundred times higher levels in healthy male ejaculates compared to infertile patients [60–63]. Our AEA (5.620 ×10^− 10^ M) and 2-AG (5.880×10^− 9^ M) secretion profiles of hiPSC-derived hSSCs are higher than reported in literature. The 2-AG secretory performance of the hiPSC-derived hSSCs reflects a healthy status and similar to that of fertile male ejaculate sperms in clinical studies.

This study quantitatively mapped the distribution and the expression profile of endocannabinoid receptors *CB1R*,* CB2R*,* TRPV1* and *GPR55* in hDFs, hDF-derived hiPSCs and hiPSC-derived hSSCs germ stem cell populations. We report a proportionally higher cannabinoid receptor content in hSSCs compared to hiPSCs and in hiPSCs compared to hDF. All cells exhibited higher expression of CB2R than of CB1R, TRPV1 and GPR55. We further report the expression of *CB1R* to be 4.07-fold, *CB2R* 3.02-fold, *TRPV1* 2.26-fold and *GPR55* 3.75-fold relative to *GAPDH* by qPCR and around 90% homogeneous positivity for all receptors on hiPSCs. Previous studies have identified transcript levels of cannabinoid receptors in different mESC lines (D3-ES, Rosa ES, E14, H18, R1, USP1) by RT-PCR ranging from very low to 50% for *Cb1r*, very low to 40% for *Cb2r*, and 80% for *Trpv1* compared to mouse brain or *Actb* as controls [17, 18, 20]. Western blot revealed conflicting protein expression levels for *CB1R* in two studies, and a single study reported a very low *CB2R* protein expression for mESCs compared to *actb* [19, 20]. The presence of *CB1R*,* CB2R*,* TRPV1* and *GPR55* was not reported in hESCs and hiPSCs in previous studies. Our quantitative mRNA and protein-based data reveal for the first time the presence of the CB1R, CB2R, TRPV1, GPR55 in hDF-derived hiPSCs at very high levels together with active secretion of endogenous cannabinoids AEA, 2-AG. Our study further reports a higher expression of *CB2R* in hiPSCs with a higher 2-AG production. Previous reports do not report the proportions of receptor subtypes since the expression levels are generally low or the employed methods are too limited for quantitation. We showed that the expression of all cannabinoid receptors in hiPSCs were higher than in hDFs. mESCs express higher CB1R and CB2R protein levels, but lower mRNA levels of *CB1R*,* CB2R* and *TRPV1* compared to MEFs [17]. Our hiPSC data clearly reveal a dominant *CB2R* expression pattern following reprogramming from hDFs. We further describe induction of expression of *CB1R* to be 2.19-fold, *CB2R* 26.38-fold, *TRPV* 0.22-fold and *GPR55* 0.24-fold, relative to *GAPDH* in hiPSC-derived hSSCs by qPCR, and we showed a homogeneously high and diffuse cellular distribution of all specific receptors, ranging from 70 to 92% with CB2R displaying the highest expression of 92% in hiPSC-derived hSSCs. Human fetal testis homogenates in the seventh week express slightly higher *CB1R* than *CB2R*, which remains remarkably stable between weeks 6 to 17 by RNA-seq [29]. Both *CB1R* and *CB2Rs* are located at LIN28 + male germ cells presenting hSSC stage [64] within the fetal testis in week 17 [29]. The pachytene spermatocytes and round spermatids of human healthy adult testes expressed the *CB1* as 0.0007-fold and 0.0006-fold, its isoform *CB1A* as 0.0008- fold and 0.0006- fold, *CB1B* as 0.00002-fold and 0.00003- fold, *CB2A* as 0.002-fold and 0.0005- fold higher when compared to housekeeping gene RPLP0 by qPCR [28]. Primary spermatocytes, pachytene spermatocytes showed nuclear CB1R, whereas the spermatogonia, late spermatocytes and round spermatids displayed cytoplasmic CB2R by immunohistochemistry [28]. Our results on the CB2R dominant CB receptor distribution on hiPSC-derived hSSCs are in line with previous data on human fetal and adult testicular spermatogonial cells and extended the endocannabinoid system involvement in hiPSC-derived hSSCs. Both CB1 and CB2 receptors presented increased expression (with a higher induction of CB2R compared to CB1R) in fertile male mature sperm compared to primary infertile patients, aged 20–45 [27]. Combining the previous literature and our present study, overall data suggests CB2R is a strong candidate for fertility induction that could possibly increase the yield of autologous hiPSC-derived hSSCs. Thus, the following work packages assessed the efficiency of CB agonists with particular attention to CB2R activation for the enrichment of hSSCs from hiPSCs.

We further report that *CB1R* and *CB2R* endogenous agonists AEA (2.515 × 10^− 6^ M) and 2-AG (3.411 × 10^− 5^ M) promote the proliferation of hiPSCs at high submaximal doses between 138 and 186 h. AEA and 2-AG make the vast majority of endogenous cannabinoids and target CB1R, CB2R, TRPV1 and GPR55 [65] but have a low affinity for CB1R (AEA has K_i_=89 nM and 2 AG has K_i_=472 nM affinity) and CB2R (AEA has K_i_=371 nM and 2 AG has K_i_=6.94 nM affinity) [25, 66]. Our effective dose window aligns with high K_i_ values of AEA and 2-AG for both receptors. The selective synthetic CB1R agonist ACPA (2.234 × 10^− 5^ M) and ML184 (2.015 × 10^− 6^ M) decreased the proliferation rate of hiPSCs between 24 and 186 h that reveals CB1R (ACPA has Ki = 2.2 nM affinity) and GPR55 (ML184 has K_i_=250 nM affinity) agonism does not support hiPSC proliferation. This is in line with our previous work that suggests CB2, but not CB1 and GPR55 agonistic pathways for the hiPSCs. The TRPV1 (CSP has K_i_=650 nM affinity) selective agonist CSP (4.318 × 10^− 7^ M) presents a late stimulation of hiPSC proliferation between 144 and 186 h that could be attributed to a limited density of active TRPV1 receptors within the hiPSC colonies. In our culture protocol, hiPSC colonies are harvested regularly every 3 or 4 days since a spontaneous differentiation frequently occurs before 144 h. As a result, the therapeutic window for TRPV1 is not appropriate for the hiPSC standard culture protocol [67, 68]. The CB2R selective agonist CB65 (2.092 × 10^− 8^ M) increased the proliferation of hiPSCs between 78 and 174 h.

CB65 exhibited an early and powerful induction of proliferation of hiPSCs with a EC_50_ value of 2.092 × 10^− 8^ M between 78 and 174 h at a 10-time lower dose compared to CPS. Furthermore, AM630, a CB2R antagonist, showed the potency and the specificity of CB65 by revealing the maintenance of hiPSC pluripotency. The synthetic AEA analog Met-AEA and 2-AG support *Oct4* and *Nanog* gene expression, marking stemness in mESCs with no effect on the proliferation rate of these cells, when applied at a dose of 10^− 12^-10^− 6^ M [17]. The CB2R agonist JWH015 (K_i_=13.8 nM for CB2R) increased the survival rate of mESCs at 2 × 10^− 7^ M with a higher potency when compared to a non-specific agonist WIN 55212-2 (K_i_=3.3 nM for CB2R, K_i_= 62.3 nM for CB1R) at 10^− 8^ M along with the induction of EB formation [18] which was attributed to the higher expression of CB2R by mESCs compared to CB1R [18]. The psychoactive pan agonist Δ^9^-THC (K_i_=7.2 nM for CB1R, K_i_=7.1 nM for CB2R) induced mESC proliferation in a dose range between 10^− 8^-10^− 4^ M by 1.5-2.5-fold compared to vehicle control [19, 20]. In the present study CB65 shows a higher affinity and thus, a superior potency at a ten times lower dose on hiPSCs when compared to JWH015 and WIN 55212-2 on mESCs. However, Δ^9^-THC is not transferrable to the ART clinic due to legal restrictions. No previous study has reported on ECS involvement and CB profiles of hiPSCs. Taken together, the 1.5-9-fold higher *CB2R* expression profile of hiPSCs compared to *CB1R*,* TRPV1* and *GPR55*, along with the 10-fold higher 2-AG secretion, when compared to AEA, suggests this may be a promising tool for to effectively obtain high yields of hSSCs from hDF-derived hiPSCs. At this point, CB65 might be a better candidate compared to 2-AG in terms of its specificity and the high affinity for CB2R.

Next, we demonstrate that CB65 increases the percentage of hSSCs in G_2_/M to 34–36% on day 10 and hSSCs in G_0_/G_1_ to 36–38% on day 12 along with an increase in ID4 + SSCs, PLZF + SPSCs by 10% on day 10 and an increase in expression of *SCP3* by meiotic spermatocytes by 1.5-fold on day 12. The non-psychoactive CB1R/CB2R antagonist and TRPV1 agonist cannabidiol (antagonist for CB1R, CB2R; agonist for TRPV1) and Δ9-THC (K_i_=7.2 nM for CB1R, K_i_=7.1 nM for CB2R) at 10^− 5^ M slightly (not statistical significant) induce DAZL+, LIN28A+, POU5F1 + hSSCs and SCP3+, STRA8+, REC8 + meiotic spermatogenic cells by 1.0- 1.25-fold more than vehicle controls on day 2 when applied to testicular air-liquid interphase (ALI) cultures from a series of middle-aged adult postmortem organ donors by qPCR. LIN28A + and POU5F1 + marked SSPCs similar to PLZF, and DAZL is expressed by spermatogenic cells from the spermatogonia to the spermatocyte stage [30]. Thus, our 10-day *PLZF* and 12-day *SCP3* expression data are in line with the 2-day expression observed in the adult testicular ALI setup. Although, Δ^9^-THC and its non-psychoactive cannabidiol presenting both pan agonists of ECS supported already ongoing spermatogenesis in adult testicles *ex vivo* at a 1000 times higher dose when compared to CB65 in the hiPSC-derived hSSCs monolayer culture platform. The synthetic CB2R agonist JWH133 (K_i_= 7.2 nM for CB2R affinity) at 10^− 6^ M increases prepubertal mouse TRA98 + spermatogonia by 10-fold, SCP3 + zygotene spermatids by 10-fold, SCP3 + pachytene spermatids by 1.5-fold and CREM + round spermatid 7-fold on day 30 in a testicular ALI culture platform, when compared to adult testicular strips excises from 30-day old adult mice [22]. CB65 provided a similar induction on SCP3 + meiotic human spermatids but not on the CREM equivalent ACR + round/elongated spermatids on day 12, at a significantly (100 times) lower dose compared to JWH133 due to a better affinity index. Taken together, CB65 significantly enhances the yield of hiPSC-derived hSSCs by inducing the mitotic G_2_/M phase of the cell cycle on day 10 that coincides with the meiotic induction of human spermatogonial stem/progenitor cells confirmed by a significantly increased number of SCP3 + haploid spermatocytes and a slightly decreased mitotic G_2_/M phase in hSSCs. Since the number of obtained haploid germ cells is very limited (varying from 0 to 14%) [16, 37, 38, 69] in literature, any improvement in number matters in the infertility clinic [16, 37, 69]. Therefore, the 10% yield in hSSCs production after CB65 treatment is a promising start and provides a base for further translational studies.

The output of ECS-induced hSSCs derivation is limited to in vitro conditions requiring further confirmation for functional performance in long-term ex vivo preclinical setups for a safe and comprehensive translation to the infertility clinic. In line with the translation to the clinic, at least three hiPSC lines should be derived from paternal human somatic cells to confirm the reproducibility and safety of the protocols. Here, we effectively reprogrammed hiPSCs from hDF, that display overall characteristic basic features of pluripotency. Subsequently, we confirmed the pluripotency through verification of protein and mRNA levels. The hiPSCs were reprogrammed from a dermal fibroblast cell line derived from a healthy donor. It has been previously shown that chromosomal abnormalities in pluripotent stem cells do not significantly impact their morphology, pluripotency, or differentiation potential [70–72]. In addition, although a normal karyotype is critical to determine the suitability of the hiPSCs for clinical applications, at this point our study is merely a “proof-of-principle” showing the enormous potential of these cells. Therefore, at this point our study did not contain karyotyping analysis. However, for clinical translation and future scale-up studies, we believe quality control of the generated hiPSCs should be supported by karyotyping data and single cell genome sequencing to evaluate possibly harmful genetic mutations and heterogeneity. In addition, we were not able to use a positive control, such as embryonic stem cells. Instead, we used the original hDFs are a negative control for pluripotent markers for our tests. Similar to ours, other preliminary hiPSC-based studies based on a single line have been widely used without positive human embryonic stem cell controls [33, 73, 74]. Generation of further germ cell differentiation steps could produce elongated functional sperms. Current limitations are mainly due to the challenging long-term maintenance of hiPSC-derived hSSCs in the monolayer culture setup. Therefore, our current data provide an improvement for the enrichment of hiPSC-derived hSSCs and hSSPCs. Future hiPSC based-trials should avoid the use of xenogeneic products and animal models and need to model the 3D human testicular niche ex vivo, benefiting from microfluidic [7, 11, 75] and organoid technologies [9, 11, 76] for long term cultures. Additionally, evaluating the obtained hSSCs through gene expression arrays or even at the single-cell level may improve the enrichment protocols. However, this study is the first to assess the role of ECS in hiPSCs and hiPSC-derived hSSCs and may allow the development of a tool for personalized treatment of male infertility in germ cell aplasia patients by using patient-derived cells. On the other hand, the hiPSC-derived hSSCs present the only autologous source for azoospermic males, and only a few research groups, including ours, have been working on protocols that would yield hSSCs in sufficient numbers to aid these patients. However, the yield of hiPSC-derived male germ cells is still limited to a mere 2–5% [11, 16]. This report demonstrates the ECS acts via CB2R agonism in hiPSCs and may enable the procurement of hiPSC-derived hSSCs at a therapeutic level. Our data are reliable in terms of normal distribution and statical relevance, confirming the pluripotent and spermatogenic cellular identities in all work packages by checking the stage-specific markers at protein and mRNA levels. Furthermore, we mapped for the first time all ECS members present in hDF-derived pluripotent and spermatogenic cells by quantitatively assessing the ligands, receptors and secretion patterns and setting a selective, agonistic, efficient therapeutic window for CB65. This synthetic cannabinoid agonist with a high affinity to CB2R fitted perfectly in the hiPSC derivation protocol by acting at an early interval to enhance the yield of hSSCs. The RTCA system was used to determine the EC_50_ of CB65 at a cellular level [40, 77]. While endpoint proliferation methods such as MTT are widely used for additional confirmation of proliferation data, they provide only a limited data on cellular activity at a specific time point. In contrast, RTCA provide a dynamic and continuous assessment of proliferation [78]. This broadly used technology for the development of personalized and precise cell-based or chemical therapeutics offers a new tool for hiPSC-based fertility treatment protocols by complementing techniques assessing genomic stability and safety. Further preclinical studies will also require metabolomic and functional evaluations prior to the translation into the phase trials comprising azoospermic patients with no or few SSC reserves due to different etiologies.

## Conclusion

Our study presents the first demonstration of the use of ECS induction to enhance production and yield of hiPSCs and hiPSC-derived hSSCs for the generation of human stage-specific germ cell populations for infertile male patients. Two reports mentioned 2-AG [60] and CB2R [27] as potential fertility biomarkers showing that expression in fertile male semen samples differed from infertile ones and underline the importance of assessment of the ECS in male fertility. We believe that cannabinoid-based mediators may be good candidates for autologous, personalized germ/stem cell-based fertility programs that could improve the production yield in culture protocols for MFI patients with complete germ cell aplasia.

## Electronic supplementary material

Below is the link to the electronic supplementary material.

Supplementary Material 1

## Data Availability

All data generated or analyzed during this study are included in this published article (and its supplementary information files).

## Abbreviations

ACPA: Arachidonylcyclopropylamide
ACR: Acrosin
AEA: Anandamide
ALI: Air-liquid Interphase
ANOVA: Analysis of Variance
ART: Assisted Reproductive Technology
BSA: Bovine Serum Albumin
B2M: Beta-2-Microglobulin
CBD: Cannabidiol
CB1R: Cannabinoid 1 Receptor
CB2R: Cannabinoid 2 Receptor
cDNA: Complementary Deoxyribonucleic Acid
CI: Cell Index
CSP: Capsaicin
DAZ: Deleted in Azoospermia Like
DMEM-HG: Dulbecco’s Modified Eagle’s Medium- High Glucose
DMRT1: Doublesex and Mab-3 Related Transcription Factor 1
EM: Embryonic Body
ECS: Endocannabinoid System
EC_50_: Half Maximal effective Concentration
FBS: Fetal Bovine Serum
FCM: Flow Cytometer
GAPDH: Glyceraldehyde-3-Phosphate Dehydrogenase
GDNF: Glial Cell Line-derived Neurotrophic Factor
GFRa1: GDNF Family Receptor Alpha 1
GPR55: G Protein-coupled Receptor 55
GPR125: G Protein-coupled Receptor 125
hbFGF: Human Basic Fibroblast Growth Factor
HBSS: Hank’s Balanced Salt Solution
hDFsHuman: Dermal Fibroblasts
hiPSCs: Human Induced Pluripotent Stem Cells
hSSPCs: Human Spermatogonial/Progenitor Stem Cells
hSSCs: Human Spermatogonial Stem Cells
ID4: Inhibitor of DNA Binding 4
IF: Immunofluorescence
ITS: Insulin- Transferrin- Sodium Selenite
ISSCR: International Society for Stem Cell Research
KLF4: Kruppel-like Factor 4
KSR: Knockout Serum Replacement
LC-ESI-MS/MS: Liquid Chromatography–tandem Electrospray Ionization–mass Spectrometry
LC-S/MS: Liquid Chromatography–Tandem Mass Spectrometry
MEF: Mouse Embryonic Fibroblast
mEpiLCs: Mouse Epiblast-like Cells
mESCs: Mouse Embryonic Stem Cells
MFI: Male Factor Infertility
mPGCs: Mouse Primordial Germ Cells
mRNA: Messenger Ribonucleic Acid
nM: Nanomolar
NOA: Non-obstructive Azoospermia
OCT4: Octamer- binding Transcription Factor 4
PBS: Phosphate-buffered Saline
pM: Picomolar
PLZF: Promyelocytic Leukemia Zinc-finger Gene
PSCs: Pluripotent Stem Cells
PIWIL4: Piwi Like RNA-Mediated Gene Silencing 4
qPCR: Quantitative Real- time Polymerase Chain Reaction
RA: Retinoic Acid
rhFSH: Recombinant Human Follicle-stimulating Hormone
RT: Room Temperature
RTCA: Real Time Cell Analysis
SCF: Stem Cell Factor
SCP3: Synaptonemal Complex Protein 3
SeV: Sendai Viral Vector
SSEA4: Stage-specific Embryonic Antigen 4
STRA8: Stimulated by Retinoic Acid 8
SOX2: SRY-Box Transcription Factor 2
TRPV1: Transient Receptor Potential Vanilloid 1
UTF1: Undifferentiated Embryonic Cell Transcription Factor 1
VA: Vitamin A
VC: Vitamin C
VE: Vitamin E
VPA: Valproic Acid
WHO: World Health Organization
2-AG: 2-Arachidonoylglycerol
2D: Two Dimension
3D: Three Dimension
Δ^9^-THC: Delta-9-Tetrahydrocannabinol
