# Supplementary material for: Endocannabinoid system upregulates the enrichment and differentiation of human iPSC- derived spermatogonial stem cells via CB2R agonism
Source: Biol Res. 2025 Mar 12;58:13. doi: 10.1186/s40659-025-00596-4 (PMC11900634; doi:10.1186/s40659-025-00596-4)
Supplement: Supplementary file 1 — Supplementary Material 1 [file 40659_2025_596_MOESM1_ESM.docx]

**Supplemental Information**

**Endocannabinoid System Upregulates the Enrichment and Differentiation of Human iPSC- derived Spermatogonial Stem Cell via CB2R Agonism**

Merve Gizer ^1,2^, Selin Önen ^2^, Özgür Doğuş Erol ^1,3,4^, Fatima Aerts Kaya ^1,3,4,5^, Tuba Reçber^6^, Emirhan Nemutlu^6^, Petek Korkusuz ^2,7^ *

^1^ Department of Stem Cell Sciences, Graduate School of Health Sciences, Hacettepe University, Ankara, 06100, Turkey

^2^ METU MEMS Center, Ankara, 06530, Turkey

^3^ Center for Stem Cell Research and Development (PEDI-STEM), Hacettepe University, Ankara, 06100, Turkey

^4^ Hacettepe University Advanced Technologies Application and Research Center (HÜNİTEK), Ankara, Turkey

^5^ Hacettepe University Laboratory Animals Research and Research Center (HÜDHAM), Ankara, Turkey

^6^ Department of Analytical Chemistry, Faculty of Pharmacy, Hacettepe University, 06100 Sıhhiye, Ankara, Turkey

^7^ Department of Histology and Embryology, Faculty of Medicine, Hacettepe University, Ankara, 06100, Turkey

*Corresponding Author: Petek Korkusuz, MD, PhD

Address: Department of Histology and Embryology, Faculty of Medicine, Hacettepe University, Sihhiye, 06100, Ankara, Turkey

E-mail: petek@hacettepe.edu.tr

Phone: +90-312-3052165

Mobile: +90-532-2968138

ORCID ID: 0000-0002-7553-3915

**Supplemental Table 1.** The antibodies that were used in this study were listed.

|  | Target Protein | Host | Dilution | Brand/ Catalog No | Application in This Study |
| --- | --- | --- | --- | --- | --- |
| Primary Antibody | Anti- Human OCT4 | Rabbit | *1:200* | Abcam/ab19857 | FCM, IF |
|  | Anti- Human SOX2 | Rabbit | *1:200* | Abcam/ab97959 | FCM, IF |
|  | Anti- Human Nanog | Rabbit | *1:200* | Abcam/ab109250 | FCM, IF |
|  | Anti- Human TRA-1-60 | Mouse | *1:200* | Abcam/ab16288 | FCM, IF |
|  | Anti- Human SSEA4 | Mouse | *1:200* | Abcam/ab16287 | FCM, IF |
|  | Anti- Human ID4 | Rabbit | *1:200* | Invitrogen/PA5-26976 | FCM, IF |
|  | Anti- Human PLZF | Rabbit | *1:200* | Boster/A00817 | FCM, IF |
|  | Anti- Human SCP3 | Rabbit | *1:200* | Abcam/ab15093 | FCM |
|  | Anti- Human ACR | Rabbit | *1:200* | Abcam/ab203289 | FCM |
|  | Anti- Human CB1R | Rabbit | *1:200* | Abcam/ab3558 | FCM, IF |
|  | Anti- Human CB2R | Rabbit | *1:200* | Abcam/ab3561 | FCM, IF |
|  | Anti- Human TRPV1 | Rabbit | *1:200* | Invitrogen/PA1-748 | FCM, IF |
|  | Anti- Human GPR55 | Rabbit | *1:200* | Abcam/ab203663 | FCM, IF |
|  | FITC Anti-Rabbit | Goat | *1:200* | Abcam/ab7086 | FCM |
|  | Anti- Human PAX6 | Mouse | *1:200* | Biolegend/862002 | IF |
|  | Anti- Human SOX17 | Mouse | *1:200* | Biolegend/628152 | IF |
|  | Anti- Human Brachyury | Rabbit | *1:200* | Abcam/ab209665 | IF |
| Secondary Antibody | Alexa Fluor^TM^ 488 Anti- Mouse | Rabbit | *1:200* | Invitrogen/A-11059 | FCM, IF |
|  | Alexa Fluor^TM^ 488 Anti- Rabbit | Goat | *1:200* | Abcam/ ab150077 | IF |
|  | SureLight™ APC Anti-Rabbit | Goat | *1:200* | Cayman/16596 | FCM |

**Supplemental Table 2.** The primer sequences for qPCR that were used in this study were listed.

| Primer sequences | | |
| --- | --- | --- |
| OCT4 | *Forward* | GCAAAACCCGGAGGAGTC |
|  | *Reverse* | TCCCAGGGTGATCCTCTTCT |
| SOX2 | *Forward* | ATGGGTTCGGTGGTCAAGT |
|  | *Reverse* | GGAGGAAGAGGTAACCACAGG |
| NANOG | *Forward* | ATGCCTCACACGGAGACTGT |
|  | *Reverse* | CTGCAGAAGTGGGTTGTTTG |
| ID4 | *Forward* | CGGGTGGGCTACTTTTCTT |
|  | *Reverse* | AGGGAATCTTTGCTCAGTGG |
| PLZF | *Forward* | CTCCCCTTCAATCTCATCCAC |
|  | *Reverse* | CCTCCCTACTTTACCATCCCTA |
| SCP3 | *Forward* | CTTGTGGAGGAGTTGTGGAG |
|  | *Reverse* | GGCTCTCGTAAACTGATCTTCC |
| ACR | *Forward* | CAGCCACAGGTACCACACAT |
|  | *Reverse* | CCAGTCATGCACATTATTTTTGCC |
| CB1R | *Forward* | TACCTGATGTTCTGGATCGG |
|  | *Reverse* | CCATGCGGGCTTGGTCTG |
| CB2R | *Forward* | CTATCCACCTTCCTACAAAGC |
|  | *Reverse* | TGAGGCACAGCATGGAGCAG |
| TRPV1 | *Forward* | GGCTGTCTTCATCATCCTGCTGCT |
|  | *Reverse* | GTTCTTGCTCTCCTGTGCGATCTTGT |
| GPR55 | *Forward* | AGGGAAGGGTGAGGAAGAG |
|  | *Reverse* | CTCTGTGGCTGTGGTTCTTAG |
| GAPDH | *Forward* | GGTGTGAACCATGAGAAGTATGA |
|  | *Reverse* | GAGTCCTTCCACGATACCAAG |
| B2M | *Forward* | CCGTGTGAACCATGTGACTTT |
|  | *Reverse* | CCTCCATGATGCTGCTTACA |


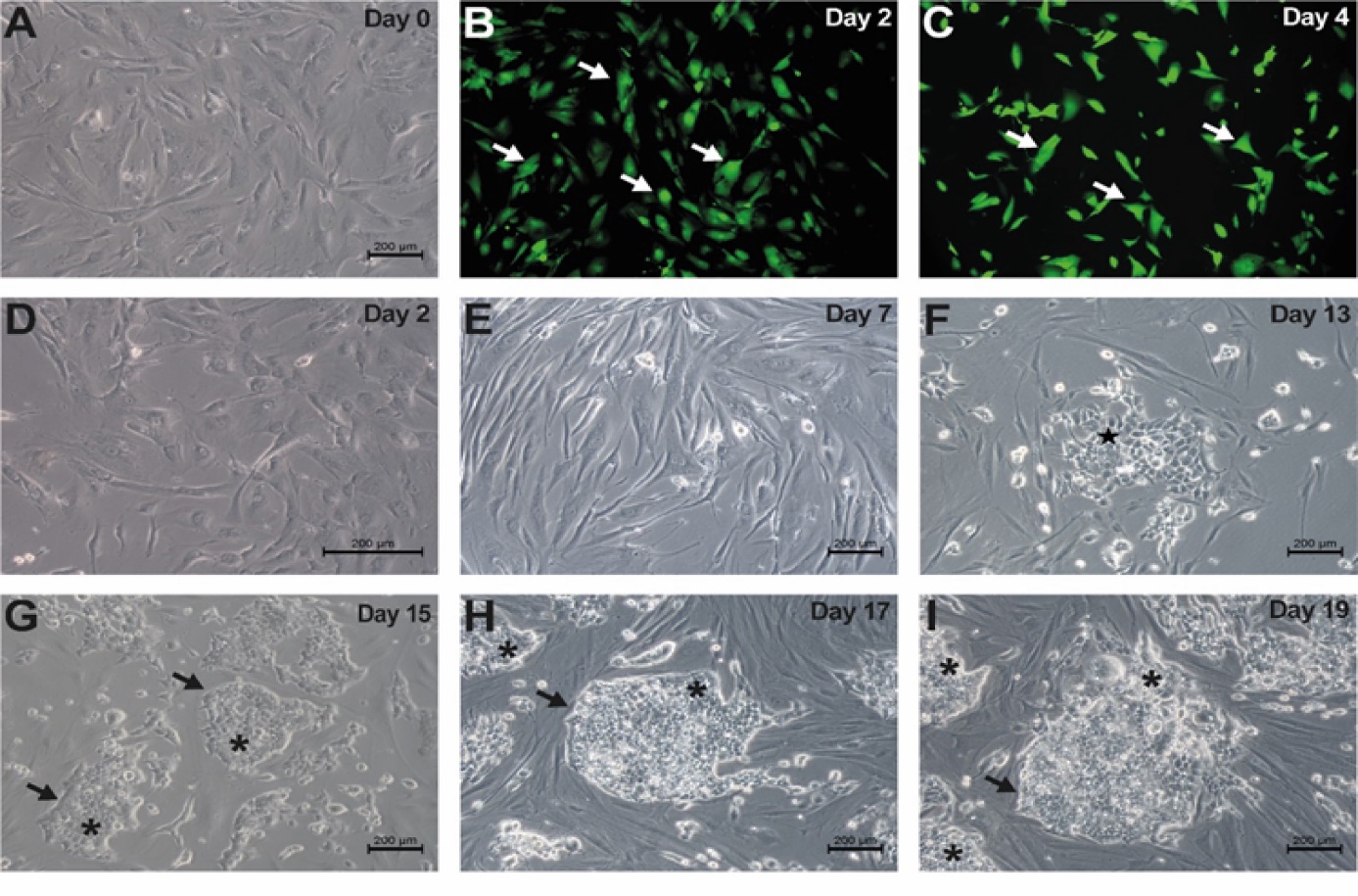


**Supplemental Figure 1** hiPSCs were re-programmed from hDFs. **a** hDFs were transfected with **b, c** Sendai virus showed GFP (*White Arrow*), **d, e** Elongated hDFs, **f** Epithelial round cell group (***) on the thirteenth day, **g** small colonies (***) with clear borders (*Black Arrow*). On the nineteenth day, **i** cells formed cubical, tightly packed, and well-defined (*Black Arrow*) hiPSC colonies (***). Scale bar is 200 µm.


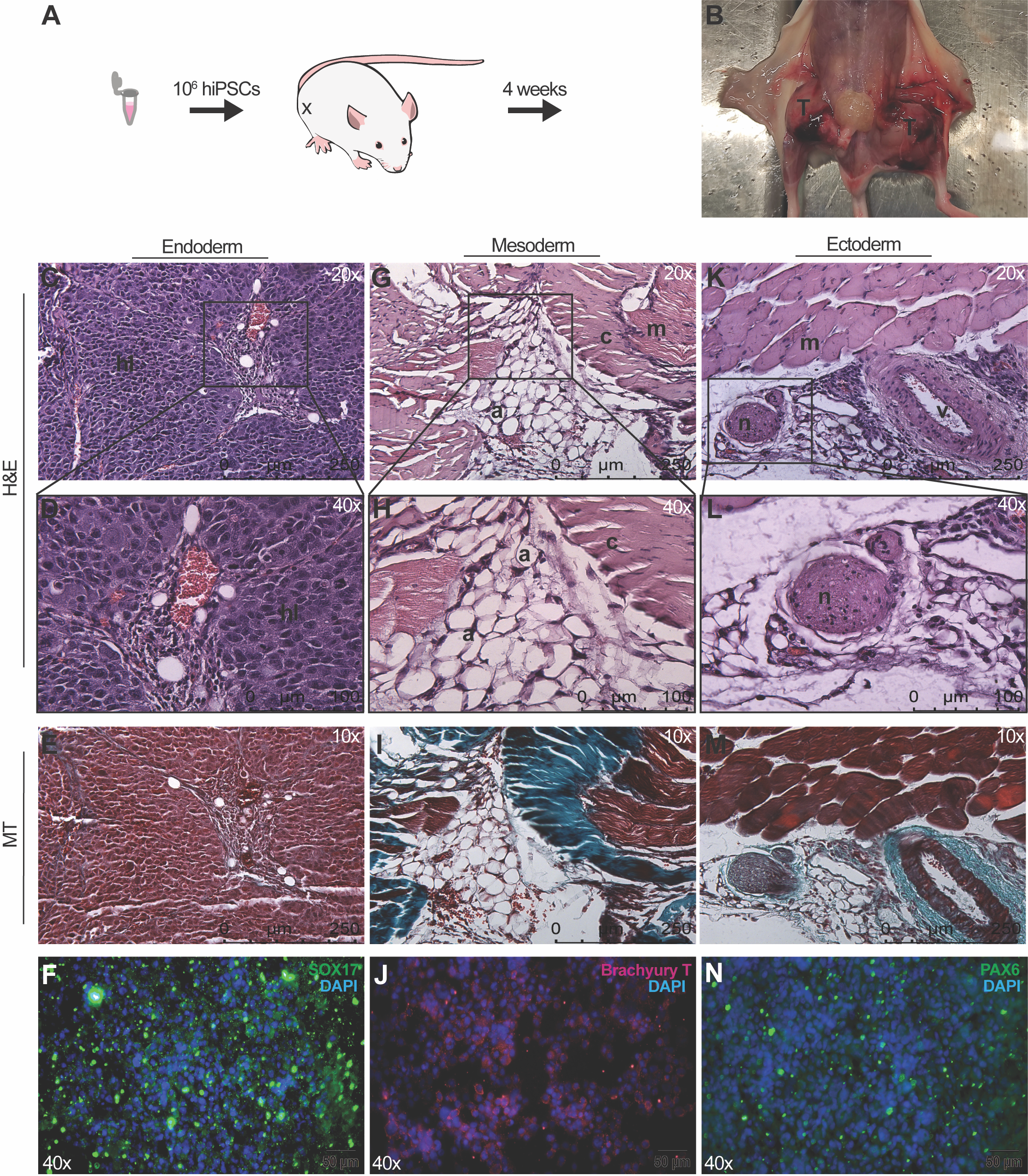


**Supplemental Figure 2.** The hiPSCs were differentiated to three- germ layers *in vivo* (A- E, G- I, K- M) and *in vitro* (F, J, N). **a** The teratoma formation assay was illustrated. **b** The hiPSCs formed teratoma (T) at the end of 4 weeks. **c – e** Liver- lobules (hl) were formed the parenchyme as an endodermal lineage. **f** The SOX17 labelled endodermal cells were differentiated from hiPSCs**. g- i** Striated muscle (m), vessel (v), adipose (a) and connective (c) tissue layers were shaped as mesodermal lineages. **j** The Brachyury T labelled mesodermal cells were obtained by differentiation of hiPSCs. **k- m** Peripheral neural- like (n) was formed as ectodermal lineage. **n** The Pax6 labelled cells were differentiated from hiPSCs. Hematoxylin- eosin (H&E), Masson’s Trichrome (MT).

**Supplemental Table 3.** Descriptive statistics of cannabinoid ligands at the supernatants of hiPSCs and hSSCs.

| **Descriptive Statistics of Endocannabinoid Ligands Levels in Supenatants (Mean±SD)** | | |
| --- | --- | --- |
|  | **AEA** | **2-AG** |
| **hiPSCs** | 7.618x10^-10^±9.511x10^-10^ M | 4.840 x10^-9^±5.225 x10^-10^ M |
| **hSSCs** | 5.620 x10^-10^±1.128 x10^-10^ M | 5.880 x10^-9^±1.182 x10^-9^ M |


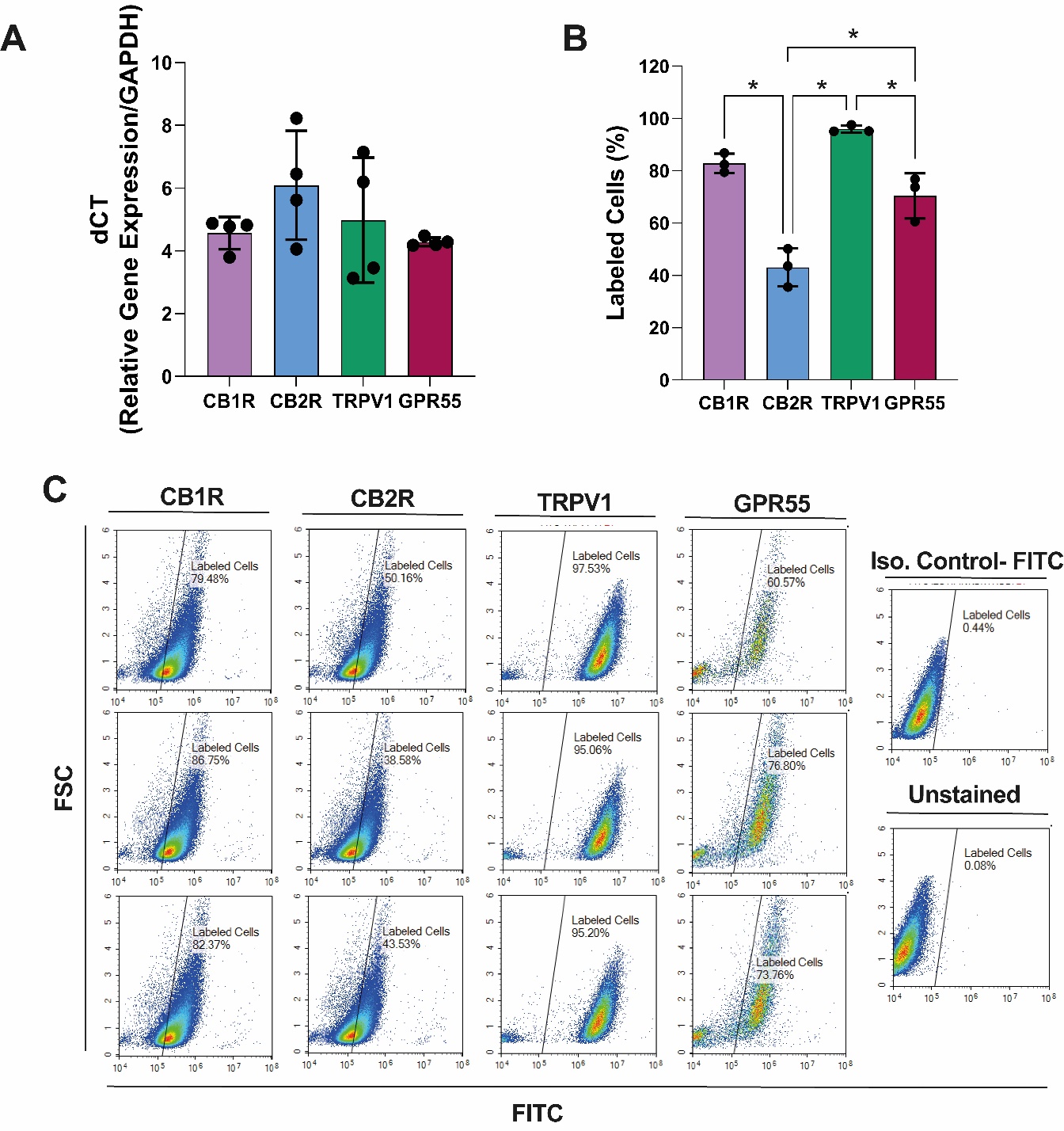


**Supplemental Figure 3** Cannabinoid system in hDFs. **a** hDFs expressed CB1R, CB2R, TRPV1 and GPR55 relative to GAPDH. **b** CB2R presented a significantly lower ratio compared to CB1R, TRPV1, and GPR55 **c** CB1R, CB2R, TRPV1, and GPR55 immune distribution in hiPSCs on a range of 79.48- 86.75%, 38.58- 50.16%, 95.06- 95.20%, and 60.57- 76.80% respectively by FCM. * p<0.05.


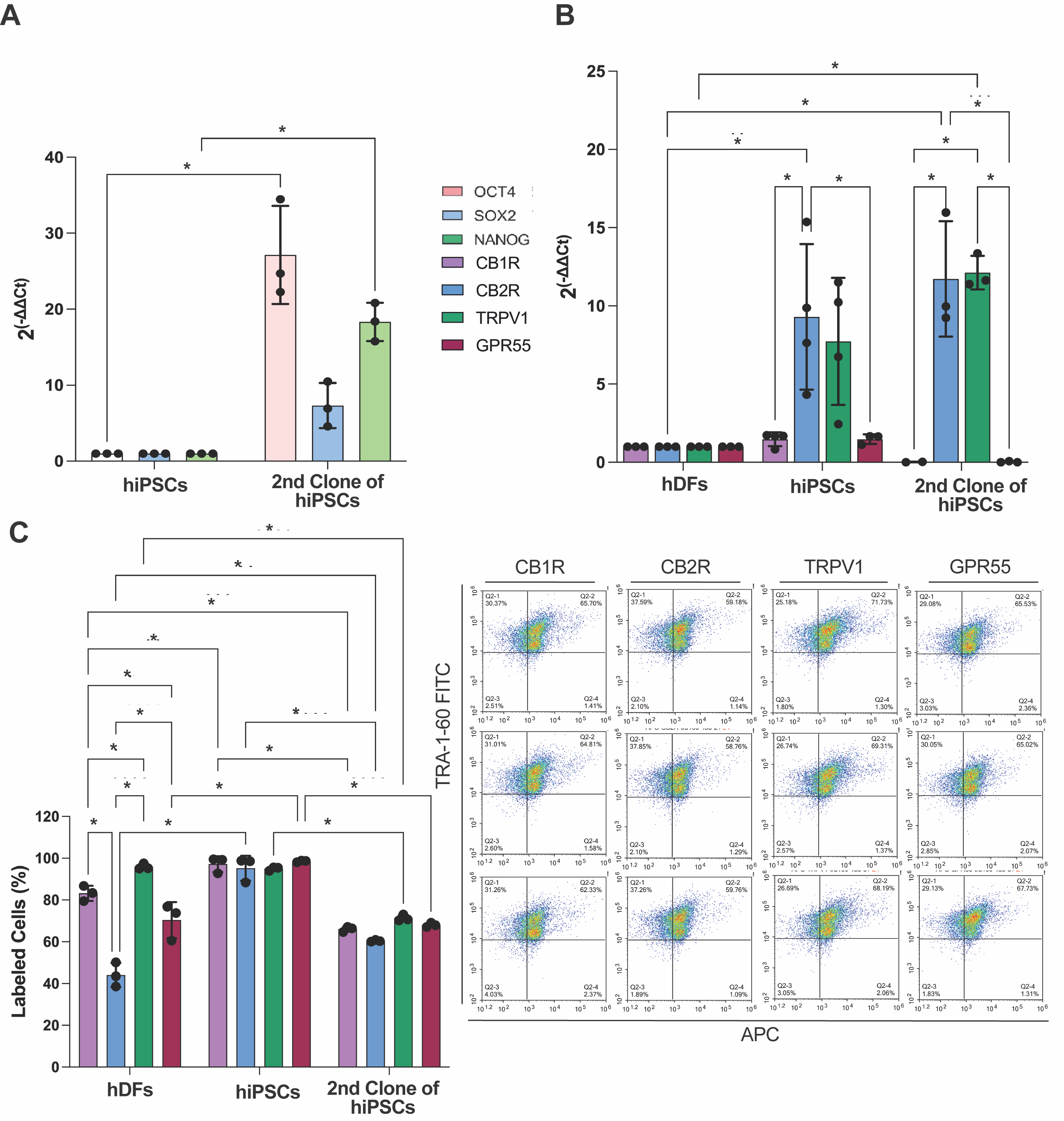


**Supplemental Figure 4.** The second clone of hiPSCs expressed pluripotency markers and endocannabinoid receptors. **a** The second clone of hiPSCs expressed OCT4, SOX2 and NANOG. **b** The second clone of hiPSCs expressed CB1R, CB2R, TRPV1 and GPR55 similar to first clone of hiPSCs. **c**  The percentages of CB1R, CB2R, TRPV1, and GPR55 labeling were slightly lower in the second clone compared to the first clone of hiPSCs.

**Supplemental Table 4.** Relative gene expression levels of CB1R, CB2R, TRPV1 and GPR55 normalized to GAPDH gene expression.

| **2 ^ΔCt^ (Relative Gene Expression/GAPDH)** | | | | |
| --- | --- | --- | --- | --- |
|  | **CB1R** | **CB2R** | **TRPV1** | **GPR55** |
| **hDFs** | 0.04500±0.01732 | 0.02250±0.02630 | 0.05500±0.05260 | 0.05000±0.008165 |
| **hiPSCs** | 0.06000±0.02000 | 0.1350 ±0.07141 | 0.2050±0.1570 | 0.1450±0.1436 |
| **hSSCs** | 2.193±0.3202 | 26.38±4.699 | 0.2250±0.07724 | 0.2467±0.01528 |

**
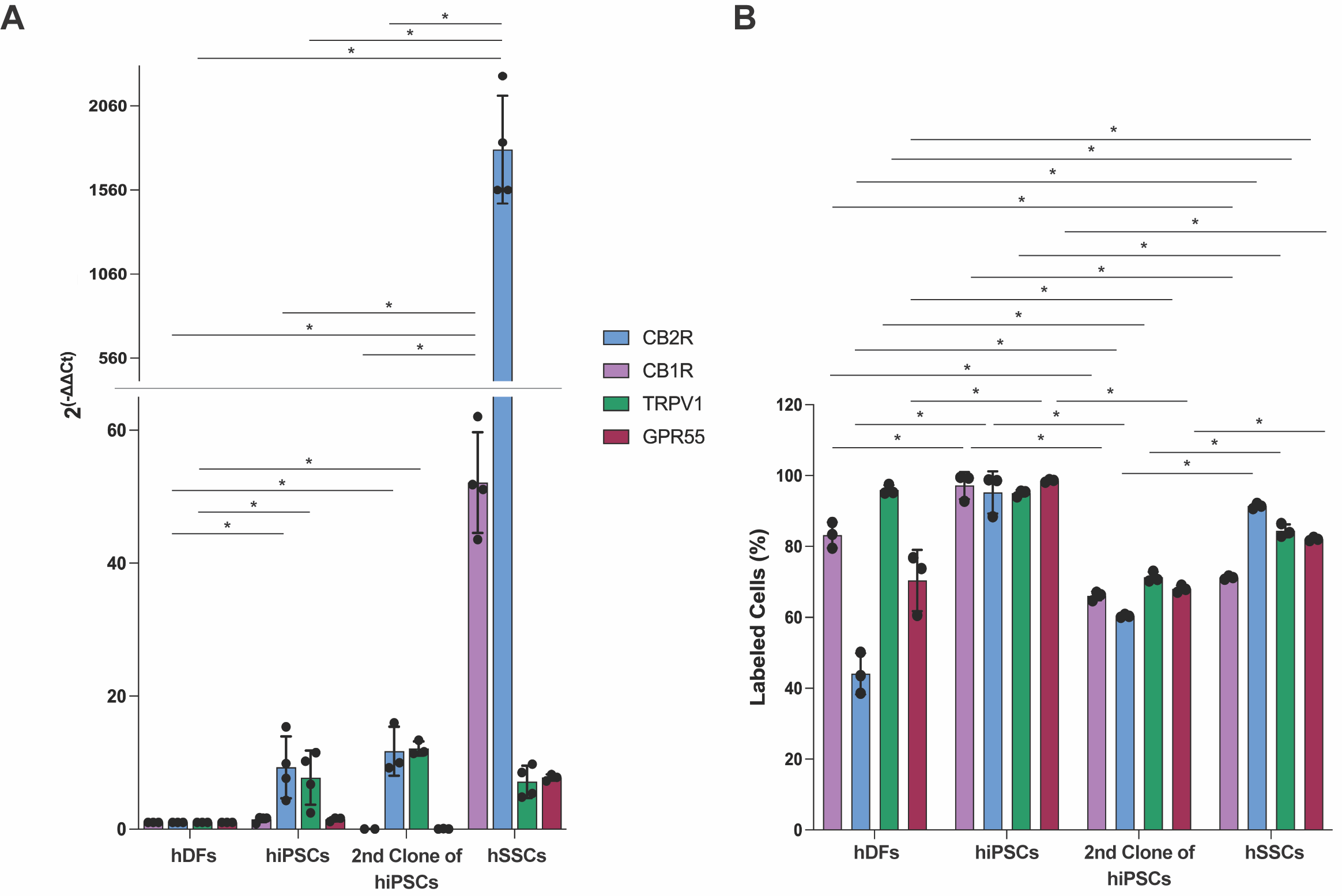
**

**Supplemental Figure 5.** Tracking of endocannabinoid system receptors thorough cellular reprograming and differentiation. **a** Expression levels of CB1R, CB2R, TRPV1 and GPR55 in hDFs, hiPSCs, second clone of hiPSCs and hSSCs relative to GAPDH by qPCR. **b** CB1R, CB2R, TRPV1, and GPR55 immune distribution in hDFs, hiPSCs, second clone of hiPSCs and hSSCs. *p<0.05.
